# Encapsulation of MSCs and GDNF in an Injectable Nanoreinforced Supramolecular Hydrogel for Brain Tissue Engineering

**DOI:** 10.1021/acs.biomac.2c00853

**Published:** 2022-10-26

**Authors:** Pablo Vicente Torres-Ortega, Rubén Del Campo-Montoya, Daniel Plano, Jacobo Paredes, Javier Aldazabal, María-Rosario Luquin, Enrique Santamaría, Carmen Sanmartín, María J. Blanco-Prieto, Elisa Garbayo

**Affiliations:** † Department of Pharmaceutical Technology and Chemistry, Faculty of Pharmacy and Nutrition, University of Navarra, C/Irunlarrea 1, 31008Pamplona, Spain; ‡ Navarra Institute for Health Research, IdiSNA, C/Irunlarrea 3, 31008Pamplona, Spain; § Tecnun, School of Engineering, University of Navarra, C/Manuel de Lardizábal 15, 20018San Sebastián, Spain; ∥ Department of Neurology and Neurosciences, Clínica Universidad de Navarra, Pamplona, C/Pío XII 36, 31008Pamplona, Spain; ⊥ Clinical Neuroproteomics Unit, Navarrabiomed, Hospital Universitario de Navarra (HUN), Universidad Pública de Navarra (UPNA), Instituto de Investigación Sanitaria de Navarra (IdisNa), 31008Pamplona, Spain

## Abstract

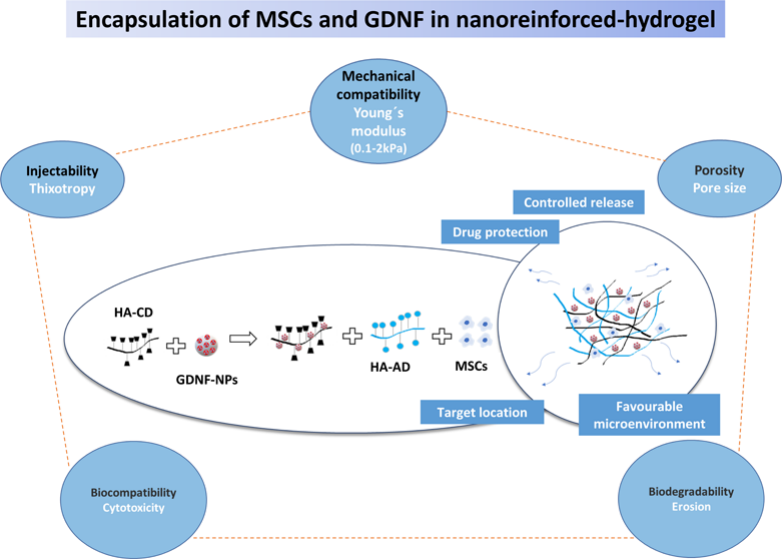

The co-administration of glial cell line-derived neurotrophic factor (GDNF) and mesenchymal stem cells (MSCs) in hydrogels (HGs) has emerged as a powerful strategy to enhance the efficient integration of transplanted cells in Parkinson’s disease (PD). This strategy could be improved by controlling the cellular microenvironment and biomolecule release and better mimicking the complex properties of the brain tissue. Here, we develop and characterize a drug delivery system for brain repair where MSCs and GDNF are included in a nanoparticle-modified supramolecular guest–host HA HG. In this system, the nanoparticles act as both carriers for the GDNF and active physical crosslinkers of the HG. The multifunctional HG is mechanically compatible with brain tissue and easily injectable. It also protects GDNF from degradation and achieves its controlled release over time. The cytocompatibility studies show that the developed biomaterial provides a friendly environment for MSCs and presents good compatibility with PC12 cells. Finally, using RNA-sequencing (RNA-seq), we investigated how the three-dimensional (3D) environment, provided by the nanostructured HG, impacted the encapsulated cells. The transcriptome analysis supports the beneficial effect of including MSCs in the nanoreinforced HG. An enhancement in the anti-inflammatory effect of MSCs was observed, as well as a differentiation of the MSCs toward a neuron-like cell type. In summary, the suitable strength, excellent self-healing properties, good biocompatibility, and ability to boost MSC regenerative potential make this nanoreinforced HG a good candidate for drug and cell administration to the brain.

## 1 Introduction

Parkinson’s disease (PD) is a progressive neurodegenerative disorder that affects approximately 1% of the population over the age of 65.^1^ Histopathologically, it is characterized by the loss of dopaminergic neurons in the *substantia nigra pars compacta* followed by a depletion of dopamine levels in the striatum and by the presence of intracytoplasmic proteins inclusions called Lewy bodies,^2^ mitochondrial dysfunction,^3^ and widespread neuroinflammation.^4^ Currently, dopamine replacement therapy is the standard treatment for PD management. However, long-term levodopa administration is often associated with significant and disabling side effects like motor and psychiatric complications as the disease progresses, leading to significant declines in the quality of life of PD patients and families.^5^ Therefore, a great challenge in the PD field is the development of novel therapies able to prevent the ongoing neurodegeneration and halt or slow down the disease progression. Cell replacement is being investigated as a therapeutic approach for neurodegenerative diseases.^6^ However, a current limitation of cell therapy is the low cell survival after implantation due to chemical/mechanical stress during culture and harvest of cells, loss of interactions between cells and the extracellular matrix (ECM), withdrawal of neurotrophic factors, and infiltration of the immune system, mainly microglia.^7,8^ The combination of cells with drug delivery systems such as hydrogels (HGs) able to supply neurotrophic factors could solve this issue and enhance the benefit of cell therapy.^9−11^

HGs are one of the most promising systems in the area of neuronal regeneration due to their tissue-like characteristics, versatility in terms of degradation, ability to release active drugs, and biocompatibility.^12^ HG-based approaches offer a promising matrix, which provides a friendly environment to deliver neurotrophic factors at the graft site, thereby increasing cell survival after transplantation.^9−11^ Moreover, HGs have demonstrated the ability to direct cell fate and change gene expression by providing stiffness and three-dimensional (3D) interactions with the cells.^13−15^ Until recently, most of the developed HGs were based on the chemical crosslinking of their polymers through covalent reactions. Although the resulting HGs may be stiffer and more stable than others obtained by different means, their crosslinks are usually irreversible, limiting less invasive routes of administration such as high Gauge syringes and catheters.^16−18^ Also, the crosslinking process may need toxic chemicals or hard conditions that could damage the potential cargo such as cells or proteins and reduce biocompatibility.^19^ As a result, in recent years, several supramolecular HGs based on the physical crosslink of their polymers by different methods such as guest–host complexes, hydrophobic interactions, and polymer-nanoparticle HGs, among other methods, have been developed.^19,20^

However, to further advance in this field, the incorporation of biologically relevant tissue complexity into these strategies is needed.^21^ The integration of nano- and microtechnologies into HG-based strategies could be advantageous for controlling the cellular microenvironment and biomolecule release, and better mimicking the complex properties of the brain tissue. Among natural polymers, hyaluronic acid (HA) is a particularly attractive material for biomedical applications, and more specifically for brain tissue applications. First, it is one of the major components of the extracellular matrix, being present throughout the body, and presents low stiffness, making biocompatibility more feasible.^22−24^ HA also exerts an anti-inflammatory effect (only high-molecular-weight HA) and a pro-survival effect on cells through its interaction with CD44, both properties being highly beneficial for cell engraftments.^25^ Several HA-based treatments have already been approved by the FDA.^26^ However, HA degrades relatively quickly, in part because of its low stiffness, possibly hampering the delivery of protein and/or cell engraftments.^27^

HA-HGs could benefit from an enhancement of their rheological properties, by chemical modifications, guest–host interactions, or nanoreinforcement, obtaining a stiffer material that degrades more slowly, while maintaining its biocompatibility.^18,28,29^ At present, several HGs have been investigated in the context of PD for the encapsulation of different drugs and also the delivery of neurotrophic factors alone or in combination with stem cells.^9−11^ In this study, we develop and characterize a multifunctional drug delivery system for brain tissue engineering, where mesenchymal stem cells (MSCs) and glial cell line-derived neurotrophic factor (GDNF) are combined into a guest–host supramolecular nanoreinforced HG for their simultaneous administration. Nanoparticles (NPs) were incorporated with a dual purpose: (1) to mechanically reinforce the HG, through hydrophobic interactions between the nanoparticles and the polymers of the HG and/or through energetically favorable adsorption of the nanoparticles on the HG^30−32^ and (2) to ensure protection and sustained release of the entrapped GDNF. Then, the effect of culturing MSCs within the nanostructured HG on the transcriptome of the stem cells was evaluated. The suitable strength, excellent shear-thinning and self-healing properties, good biocompatibility, and ability to boost MSC regenerative potential make this supramolecular nanoreinforced HG a good candidate for drug and cell administration to the brain.

## 2 Experimental Section/Methods

### 2.1 Preparation of Supramolecular Guest–Host HA-Based HGs

The protocol for HA-HG preparation was adapted from previous work.^28,33^ All chemicals were purchased from Sigma-Aldrich (St.Louis, MO) unless otherwise stated.

#### 2.1.1 HA-CD Synthesis

##### 2.1.1.1 Synthesis of *p*-Toluensulfonic Anhydride (Ts_2_O)

*p*-Toluenesulfonyl chloride (16 g) and *p*-toluenesulfonic acid monohydrate (4 g) were dispersed in methylene chloride (100 mL) (Scharlab, Barcelona, Spain). The reaction mixture was stirred overnight and filtered to remove the unreacted *p*-toluensulfonyl chloride. The filtrate solution was evaporated, and then the solid obtained was dried with vacuum overnight.

##### 2.1.1.2 Synthesis of 6-*O*-Monotosyl-6-deoxy-β-cyclodextrin (CD-Tos)

β-Cyclodextrin (CD) (22.39 g) and Ts_2_O (9.42 g) were dispersed in deionized water (200 mL), and the suspension was stirred for at least 2 h. Then, NaOH (100 mL, 2.5 M) (Scharlab, Barcelona, Spain) was added to the reaction mixture. The unreacted Ts_2_O was removed by filtration, and the solution was acidified by the addition of HCl (37%) (Scharlab, Barcelona, Spain) until pH 2. The precipitate was filtered under vacuum, and the solid obtained was washed with diethylether (3 × 25 mL) (Scharlab, Barcelona, Spain).

##### 2.1.1.3 Synthesis of HA-CD

Sodium hyaluronate (1 g, *M*_w_: 1000–1500 kDa) (Fagron, Barcelona, Spain) was dissolved in deionized water (500 mL) at 80 °C. The solution was left under continuous stirring overnight. Then, CD-Tos (0.13 g) was added to the solution. After 62 h, the product was frozen and lyophilized for 5 days. ^1^H NMR (with D_2_O as solvent) spectroscopy was used to confirm the synthesis of HA-CD.

#### 2.1.2 HA-AD Synthesis

HA (1 g) was slowly dissolved in deionized water (400 mL). Then, triethylamine (TEA) (0.26 mL) and adamantane (AD) (0.37 g) were added to the solution. The reaction mixture was stirred for 62 h at room temperature (RT). Then, the solution was filtered under vacuum and lyophilized for at least 5 days. ^1^H NMR (with D_2_O as a solvent) spectroscopy was used to confirm the synthesis of HA-AD.

#### 2.1.3 Quantification of Residual TEA in HA-AD

To quantify the residual amount of TEA, quantitative ^1^H NMR was performed using a known amount of dimethylsulfone as standard. Subsequently, the ratio of the integrated signals was determined and the amount of TEA was calculated and expressed as mg TEA/mg HA-AD.

### 2.2 Preparation of GDNF-Loaded Nanospheres with TROMS

Human GDNF was expressed and purified from BHK-21 cells using a Semliki Forest virus (SFV) expressing vector as described before.^34^ GDNF-loaded NPs were prepared by solvent extraction/evaporation method using the Total Recirculation One-Machine System (TROMS). Briefly, the organic solution composed of methylene chloride/acetone (2 mL, 3:1) and Resomer RG 503H poly(lactic-*co*-glycolic acid) (PLGA) (50 mg, *M*_w_: 24,000–38,000 Da) was injected through a needle with an inner gauge diameter of 0.17 mm at a ratio of 35 mL/min into the inner aqueous phase (150 μL). The inner water phase contained GDNF (40 μg) in phosphate-buffered saline (PBS) at pH 7.4, human serum albumin (HSA) (2.5 mg) and poly(ethylene glycol) 400 (90 μL). Next, the primary emulsion (W1/O) was recirculated through the system for 1:30 min under a turbulent regime at a flow rate of 35 mL/min. The first emulsion was then injected into the external aqueous phase (W2) composed of 1% poly(vinyl alcohol) (PVA) (20 mL, *M*_w_: 13,000–23,000 Da). The turbulent injection through the needle with an inner gauge diameter of 0.17 mm resulted in the formation of a multiple emulsion (W1/O/W2), which was further homogenized by circulation through the system for 5 min. The W1/O/W2 emulsion was stirred at 300 rpm at RT for at least 2 h to allow solvent evaporation and nanospheres formation. Non-loaded NPs (without GDNF) were prepared following the same method described above.

### 2.3 Characterization of Nanospheres

#### 2.3.1 Particle Size Analysis

The mean particle size was determined by Nanoparticle Tracking Analysis (NTA) (Nanosight NS300, Malvern Instruments). All samples were diluted in Milli-Q Water (0.5 mL). The ideal measurement concentrations were found by pretesting the ideal particle per frame value (20–100 particles/frame). The same samples were used to analyze the surface charge of the NPs by measuring the zeta potential with laser Doppler velocimetry (Zetasizer Nano, Malvern Instruments) at 25 °C. Measurements were performed in triplicate, and the results were presented as mean ± standard deviation.

#### 2.3.2 Drug Content

The quantity of GDNF encapsulated in the nanospheres was determined by dissolving the freeze-dried loaded particles (5 mg) in dimethyl sulfoxide (DMSO) (1 mL). The amount of GDNF was measured by ELISA using GDNF enzyme-linked immunosorbent assay kit from Thermo Fisher (Waltham, MA).

#### 2.3.3 *In Vitro* Release

To characterize the *in vitro* release profile of GDNF from PLGA-NPs, the freeze-dried loaded NPs (3 mg, *n* = 3) were dissolved in PBS (150 μl) supplemented with 0.1% HSA and microbiologically protected with 0.02% w/w sodium azide. The incubation was performed with orbital rotation using an Eppendorf multirotator. Samples were maintained at 37 °C with orbital rotation (15 rpm) for 37 days. The times selected to perform the release profile were 6 h, 1, 2, 3, 7, 14, 20, 28, 35, and 37 days. At these times, tubes containing the samples were centrifuged (25,000*g*, 15 min) and the supernatants were aliquoted and frozen at −20 °C. Pellets were resuspended with the same volume of fresh medium. The GDNF present in the supernatants was quantified by ELISA. Moreover, considering the instability of GNDF and its possible degradation on the release medium, the data extracted from supernatant quantification were confirmed by measuring the remaining content of GDNF within NPs. For this, the freeze-dried loaded NPs (3 mg) were dissolved in the above medium and incubated in the same conditions. At 1, 2, 7, and 14 days, samples were centrifuged and the GDNF entrapped in the pellets was extracted with DMSO. Finally, the GDNF present in the pellets was quantified by ELISA. The experimental data obtained were tested for agreement with the power law release model proposed by Ritger–Peppas^35^
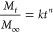
1where  is the total fraction of drug released, *k* is a constant incorporating characteristics of the macromolecular network system and the drug, *t* is the time (in days), and *n* is the diffusional exponent.

#### 2.3.4 Determination of Residual PVA

The residual amount of PVA associated with NPs was quantified by a colorimetric method based on the formation of a blue complex between two adjacent hydroxylic groups in the chain of PVA and an iodine molecule.^36^ Briefly, the freeze-dried NPs (3 mg) were treated with NaOH (2 mL, 0.5 M) and incubated for 20 min at 40 °C to degrade the matrix. Then, each sample was neutralized with HCl (900 μL, 1 M) and the volume was adjusted with ultrapure water (5 mL). To develop the colored complex, a boric acid solution (3 mL, 0.65 M), a solution of I_2_/KI (0.5 mL, 0.05/0.15 M), and ultrapure water (1.5 mL) were added to each sample. After 15 min of incubation at RT, the obtained solution (280 μL) was loaded in triplicate into a 96-wells plate. Finally, the absorbance intensity was determined at 690 nm using the microplate spectrophotometer iEMS Reader MF (Labsystem). The samples for the regression standard line based on known amounts of PVA were prepared from a PVA stock solution through serial 1:2 dilutions and were treated in the same manner as described above. Each dilution was loaded in triplicate in the same microplate as the samples. The results were presented as the ratio between the calculated residual amount of PVA and the real quantity (considering the yield) of the produced NPs by subtracting the content of the cryoprotectant, expressed as a percentage (%w/w, PVA/NPs).

### 2.4 Preparation of HGs (6 wt % HG, 6 wt % HG-NPs, 6 wt % HA, 6 wt % HG-MSCs, 6 wt % HG-NP-MSCs, and 6 wt % HA-MSCs)

The HGs were prepared using a polymer concentration of 6%, maintaining a stoichiometric balance of hyaluronic acid−β-cyclodextrin (HA-CD) and hyaluronic acid–adamantane (HA-AD) (1:1). The total mass of HA-CD and HA-AD for gel formation was determined based on the modification degree of each component according to the equations described by Loebel et al.^28^ The polymeric NPs were incorporated at 30% of the total composition. For 6 wt % HG, the HA-CD and HA-AD were individually dissolved in Dulbecco’s modified Eagle’s medium (DMEM) and mixed. For 6 wt % HG-NPs, NPs were mixed with HA-CD component, and then the second component (HA-AD) was incorporated. For the unmodified HG (6 wt% HA), the HA was dissolved in DMEM. The HGs were briefly centrifuged to remove entrapped air and transferred to the back of a syringe for *in vitro* characterization. MSCs were isolated from hind limbs of Sprague–Dawley rats as previously reported.^37^ Briefly, rat femurs and tibias were flushed with cell medium (DMEM supplemented with 10% fetal bovine serum) and the collected marrow was seeded into T-175 flasks for MSCs attachment. The medium was changed to eliminate non-adherent cells every 2–3 days and MSCs were expanded in T-175 flasks after 10–12 days. MSCs were cultured in DMEM supplemented with fetal bovine serum (20%), streptomycin (100 μg/mL) and penicillin (100 U/mL), and basic FGF (10 ng/mL). For cell encapsulation, the 6 wt % HG, 6 wt % HG-NPs or 6 wt % HA solutions were first included in the back of the syringe and then the cell suspension was incorporated to form the 6 wt % HG-MSCs, 6 wt % HG-NP-MSCs, and 6 wt % HA-MSCs, respectively. The blend was continuously mixed until a homogeneous HG was formed.

### 2.5 Characterization of the HGs (6 wt % HG and 6 wt % HG-NPs)

#### 2.5.1 Rheological Characterization

The rheological characterization of all HGs was performed using the rheometer (MCR 301 SN80439479) equipped with a 25 mm plate geometry and 0.850 mm gap. The amplitude sweeps were carried out from 0.1 to 100% at 25 °C and the frequency was kept at 10 s^–1^. Oscillatory frequency sweep measurements were conducted between 0.15 and 100 rad s^–1^ with a shear strain of 0.5%. The self-healing properties were carried out by a three-step cyclic strain time sweep based on other works.^38,39^ The low strain phase was performed for 0.5 s at 0.5% strain and the high strain phase was conducted for 250 ms at 100% strain. The frequency was 10 Hz for both phases.

#### 2.5.2 Determination of Young’s Modulus and Injectability Tests

The determination of Young’s modulus and injectability was performed using a universal testing machine capable of precisely controlling both the displacement of the crosshead and the force applied. The equipment used was a Zwick/Roell uniaxial testing machine model ZwickiLine Z1.0. The load cell used was a Zwick/Roell Xforce P with a maximum load of 50 N. The software to control the machine and record both the load and the displacement was the Zwich/Roell testXpert III v1.4.

##### 2.5.2.1 Young’s Modulus Measurement

A spherical indenter was used to determine the elastic modulus. During the test, a force was applied with the indenter against the sample to be studied. During this load, both the force used to “stick” the indenter and the penetration distance at each moment were recorded. Young’s modulus was determined using the theories and mathematical models developed by Hertz for spherical head indenters.^40^ The expression used to deduce this module is as follows (eq 2)
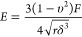
2where *E* is the Young’s modulus, υ is Poisson’s ratio, *F* is the measured force, *r* is the radius of the indenting sphere, and δ is the indented depth. In this study, a value of 0.5 was assumed for Poisson’s ratio. During all experiments conducted, it was guaranteed that both the indented depth and the sphere-sample contact area were within the limits that make this model valid. In the first stage of the experiment, the sphere was approximated to the material surface to be analyzed at a speed of 6 mm/min until it was reached an initial load of 1.5 mN. Once this preload is reached, the ball-surface contact was assumed to have been reached. After this contact, the test itself began. For this, the moving speed of the sphere was set at 6 mm/min and both the force and the penetration depth were recorded. The test ended when the reached depth was equivalent to 20% of the initial sample thickness. When the test finished, the force *vs* depth curve was obtained. This curve was adjusted to eq 2 for deducing the Young’s modulus of the sample.

##### 2.5.2.2 Injectability Tests

For the injectability tests, a setup that allows fixing a syringe vertically in the uniaxial testing machine was designed. Once loaded with the studied material, the syringe was placed in the central hole of the holder. Then, the crosshead moved downward to push the syringe plunger.

During experiments, the position of the actuator was recorded, as well as the force exerted on the plunger flange. Before starting the test, and to ensure contact between the crosshead and syringe plunger, the crosshead of the testing machine was moved downward at a speed of 50 mm/min. When reaching a 0.1 N load, the test itself began, and the load and the position of the crosshead were recorded. The force necessary to extrude the materials at different plunger speeds or flows was determined from these tests.

#### 2.5.3 *In Vitro* Degradation Test of Hybrid Systems (6 wt % HG-NPs)

The degradation behavior of the 6 wt % HG-NPs at 37 °C was characterized using two different methods. First, the 6 wt % HG-NP samples (300 μL) were introduced on plastic supports and the samples were immersed in Petri dishes with Milli-Q Water (1.5 mL). At selected times (1, 2, and 7 days), the samples were collected and freeze-dried. Then, the dried samples were weighed (W1).

The rate of residual weight (RW) was calculated as RW = W1/W0 × 100%.

The second strategy used to characterize the 6 wt % HG-NP degradation was the carbazole assay.^18,28,41^ Briefly, the HG (100 μL) was added to a 1.5 mL Eppendorf tube and centrifuged to remove any entrapped air. Then, PBS (400 μL) was added to the tube (*n* = 3). At 3, 7, 10, and 14 days, the PBS was carefully removed and collected before being replaced with fresh PBS (400 μL). On day 14, the remaining gel was disrupted using hyaluronidase (1 mg/mL) from bovine testes. The standard curve was prepared with different concentrations of glucuronic acid. The total concentration of glucuronic acid in the HGs was determined by disrupting the 6 wt % HG-NPs (100 μL) for 14 days. Then, each sample (50 μL) was added to a glass tube followed by ice-cold sodium tetraborate in concentrated sulfuric acid (1 mL, 25 × 10^–3^ M). Tubes were incubated for 10 min at 100 °C and then were cooled on ice. Next, 0.125 wt % carbazole in absolute ethanol (30 μL) was added and vortexed. The samples were then incubated for 15 min at 100 °C. Following incubation, the tubes were allowed to cool to RT and the solution (200 μL) was transferred to a 96-well plate for measurement. The absorbance at 525 nm was read using a plate reader.

#### 2.5.4 *In Vitro* Release Study of GDNF from Hybrid Systems (6 wt % HG-NPs)

The 6% (w/v) HG-NPs were prepared according to the protocol previously described and placed on 0.4 μm millicells hanging cell culture insert (*n* = 3, 200 μL each). Then, HGs were submerged in 1 mL of PBS in a 12-well plate (Corning, MA). GDNF release was measured for 14 days. At each time point (6 h, 1, 2, 3, 7, and 14 days), the millicells were transferred to another well with fresh PBS. The GDNF contained in the PBS was collected and analyzed by ELISA. The experimental data obtained were tested for agreement with the power law release model proposed by Ritger–Peppas (eq 1), detailed in Section 2.3.3.

#### 2.5.5 Bioactivity Assay of GDNF from Hybrid Systems (6 wt % HG-NPs)

For the bioactivity of GDNF released from the 6 wt % HG-NPs, the biomaterial was placed on a 0.4 μm millicell hanging cell culture insert, from which GDNF was released in a sustained manner into the PC12 cell culture. The cell density used was 2000 cells/cm^2^. After 10 days, the presence of neurites in the PC12 culture was analyzed. Furthermore, we assessed the neurite outgrowth by counting the number of cells with one or more neurites for each condition and comparing them (*n* = 6). The results were compared to non-formulated GDNF (free GDNF).

#### 2.5.6 Scanning Electron Microscopy

A Zeiss Sigma Gemini scanning electron microscope was used to characterize the 6 wt % HG, 6 wt % HG-NPs, and NPs morphology with an acceleration voltage of 10 kV and different magnifications. Samples were previously prepared, lyophilized, and coated with a few nanometers of Palladium (SC7620 Mini Sputter coater).

#### 2.5.7 *In Vitro* Cell Viability and Neural Biocompatibility

Rat adrenal PC12 cells were purchased from ATCC. Rat PC12 cells were cultured on collagen I-coated plate (Invitrogen, Waltham, MA) in Roswell Park Memorial Institute (RPMI) medium supplemented with horse heat-inactivated serum (5%), fetal bovine serum (10%), streptomycin (100 μg/mL), and penicillin (100 U/mL). MSCs and PC12 cells were incubated in a humidified atmosphere with 5% CO_2_ at 37 °C. The cytotoxicity of the biomaterial was evaluated by incubation of MSCs with the HGs extracts according to ISO 10993-5 and after encapsulating the cells in the HGs. On the other hand, the neural compatibility studies in PC12 cells were conducted by incubation of cells with the HGs extracts according to ISO 10993-5 and by surface contact of the HGs with the cells in culture.

To prepare the HG extracts, the 6 wt % HG, the hybrid system (6 wt % HG-NPs) and the 6 wt % HA without functionalization were incubated in DMEM (4 mL) culture medium with hyaluronidase (0.2 mg/mL) to extract the different components of the HGs. The samples were extracted with hyaluronidase (0.2 mg/mL) at 37 ± 1 °C for 24 ± 2 h under magnetic stirring, and then, the extracted solution was filtered through 0.22 μm millipore filters for sterilization. PrestoBlue and Live/dead assays were used to evaluate the cytotoxicity of different samples. Briefly, MSCs cells were seeded in 96-well culture plates at a density of 3 × 10^3^ cells per well. After the attachment of the cells, the culture medium was removed and the cells were treated with the HG extracts. After 24 and 72 h of incubation, PrestoBlue and Live/dead assays were performed. For PrestoBlue, the solution (10 μL) was added to each well and the plate was incubated for 3 h in the dark at 37 °C. The absorbance was measured at 570 nm as the experimental wavelength and 600 nm as the reference or normalization wavelength. The cell viability was evaluated by eq 3
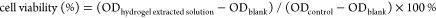
3

For Live/dead assay, cells were incubated with the reactive dye for 30 min in the dark at RT.

On the other hand, to study the toxicity after encapsulating the MSCs in the HGs, the 6 wt % HG, 6 wt % HG-NPs, or 6 wt % HA solutions were first included in the back of a syringe and then the cell suspension was incorporated to form the 6 wt % HG-MSCs, 6 wt % HG-NP-MSCs, and 6 wt % HA-MSCs, respectively. The blend was continuously mixed until a homogeneous HG was formed. The morphology and viability of encapsulated cells were analyzed using a Live/dead assay after 24 h.

Finally, to study neuronal compatibility, the PC12 cell line was treated with the extracts of the different HGs and by direct contact with the HGs without extraction. For the cytotoxicity assessment of the HGs extracts, the cells were seeded in 96-well culture plates at a density of 3 × 10^3^ cells per well. Similarly, the cells exposed to direct contact with the HGs were seeded in 24-well culture plates at a density of 2 × 10^4^ cells per well. Cells treated with the extracts were analyzed by PrestoBlue assays at 24 and 72 h and by Live/dead assay at 24 h. On the other hand, the cellular viability of the cells in contact with the different HGs was investigated using a Live/dead assay after 24 h of incubation.

### 2.6 Bulk RNA-seq Assay

2 × 10^4^ MSCs were seeded in 24-well plates embedded or not within the nanoreinforced HG. HG (100 μL) was added per well. Each well contained 1 mL of media with hyaluronidase from bovine testes (0.22 mg/mL, type I-S) (Merck KGaA, Darmstadt, Germany). Cells were cultured for one week. RNA was extracted with RNeasy Kit (Qiagen, Germany) and stored at −80 °C until further processing. Roughly 150 ng of high-quality total RNA were used for transcriptomic interrogation using Illumina’s Stranded Total RNA Prep Ligation with Ribo-Zero Plus according to the manufacturer’s instructions. Briefly, cytoplasmic and mitochondrial rRNAs as well as β-globin transcripts were depleted from the samples. The remaining RNA was fragmented and reverse-transcribed. A second strand cDNA synthesis step removed the RNA template while incorporating dUTP in place of dTTP to preserve strand specificity. Next, double-stranded cDNA was A-tailed, then ligated to Illumina anchors bearing T-overhangs. PCR amplification of the library allowed the barcoding of the samples with 10bp dual indexes and the completion of Illumina sequences for cluster generation. Libraries were quantified with Qubit dsDNA HS Assay Kit (Thermo Fisher Scientific) and their profile was examined using Agilent’s HS D1000 ScreenTape Assay (Agilent). Sequencing was carried out in an Illumina NextSeq. 2000 (Illumina) using paired-end, dual-index sequencing (Rd1: 59 cycles; i7: 10 cycles; i5: 10 cycles Rd2:59 cycles) at a depth of 50 million reads per sample.

#### 2.6.1 RNA-Seq Data Analysis

RNA-sequencing data analysis was performed using the following workflow: (1) the quality of the samples was verified using FastQC software (https://www.bioinformatics.babraham.ac.uk/projects/fastqc/); (2) the alignment of reads to the rat genome (mm10) was performed using STAR;^42^ (3) gene expression quantification using read counts of exonic gene regions was carried out with featureCounts;^43^ (4) the gene annotation reference was Gencode v27; and (5) differential expression statistical analysis was performed using R/Bioconductor.^44^

First, gene expression data were normalized with edgeR and voom.^45,46^ After quality assessment and outlier detection using R/Bioconductor,^44^ a filtering process was performed. Genes with read counts lower than 6 in more than 50% of the samples of all of the studied conditions were considered as not expressed in the experiment under study. LIMMA was used to identify the genes with significant differential expression between experimental conditions.^46^ Genes were selected as differentially expressed using a *p*-value cut-off *p* < 0.01. Further functional and clustering analyses and graphical representations were performed using R/Bioconductor and clusterProfiler.^43,47^

Functional analysis of RNA-seq outputs was performed by Metascape,^48^ applying Reactome database using default settings (minimum overlap: 3; minimum enrichment: 1.5; *p* < 0.01).

## 3 Results and Discussion

### 3.1 Synthesis of HA-CD and HA-AD

**Figure 1:**
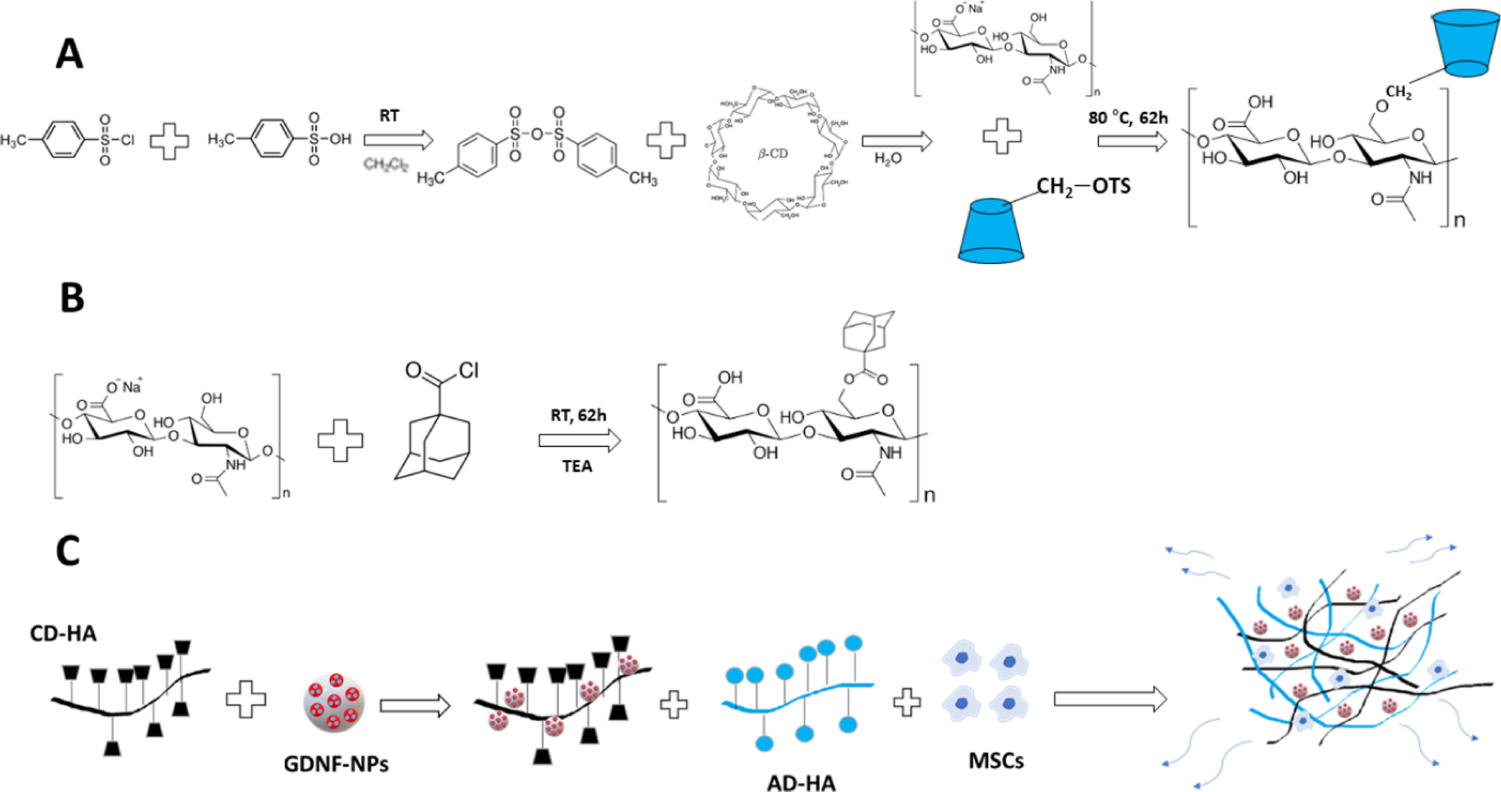
Schematic illustration showing the functionalization and synthesis of the supramolecular HA-HG, where MSCs and GDNF are combined into a nanoreinforced HA-based HG. (A) HA functionalization with CD through nucleophilic substitution. (B) HA functionalization with AD through nucleophilic acyl substitution. (C) Schematic illustration of the preparation of the hybrid system.

As shown in Figure 1, HA was functionalized with CD (host-moiety) and AD (guest-moiety) *via* nucleophilic substitution as previously reported.^28^ The specific synthetic routes of HA-CD and HA-AD are depicted in Figure 1A,B. The preparation steps of the hybrid system are represented in Figure 1C. The resultant HA-CD and HA-AD were freeze-dried and characterized by ^1^H NMR spectroscopy. HA-CD and HA-AD were recovered as a spongy white solid with yields of 86.5 ± 3.4% (*n* = 4) for HA-CD and 68.7 ± 3.8% for HA-AD (*n* = 3). Modification of HA with pendant CD (10.6 ± 1.5%) was determined by integration of the signal for the hydrogen on position 1 of CD (H1); (7 Hs, shaded blue) relative to the signal for *N*-acetyl singlet of HA (3 Hs, shaded red) (Figure S1, Supporting information). Modification of HA (40.3 ± 3.5%) with pendant AD was determined by integration of the AD hydrogens (15 Hs, shaded blue) relative to the sugar ring of HA (10 Hs, shaded red) (Figure S2. Supporting information). Herein, the total number of hydrogens (15 Hs) of the adamantane group, appearing in the ^1^H NMR spectra in the range from 2.05 to 1.60 ppm, was considered to quantify the modification of HA. This fact represents a different approach to the one used in previous reports,^28,49^ where researchers used only the methylene groups of the adamantane (12 Hs appearing in the range from 1.85 to 1.60 ppm in the ^1^H NMR). A quantitative ^1^H NMR was performed using a known amount of dimethylsulfone as standard (Figure S3, Supporting information) and the residual amount of TEA from the HA-AD synthesis was determined, resulting in 0.13 mg of TEA/mg HA-AD. In an attempt to eliminate the TEA traces trapped in our AD-modified HA, 6-day dialysis was carried out, where the water (3 L) was changed twice a day. After the lyophilization, a ^1^H NMR experiment was performed although no significant amount of TEA was eliminated. Nonetheless, this trace amount of the TEA is not considered an issue since the final HG did not show significant toxicity toward PC12 cells.

### 3.2 Preparation and Characterization of GDNF-Loaded NPs

**Figure 2:**
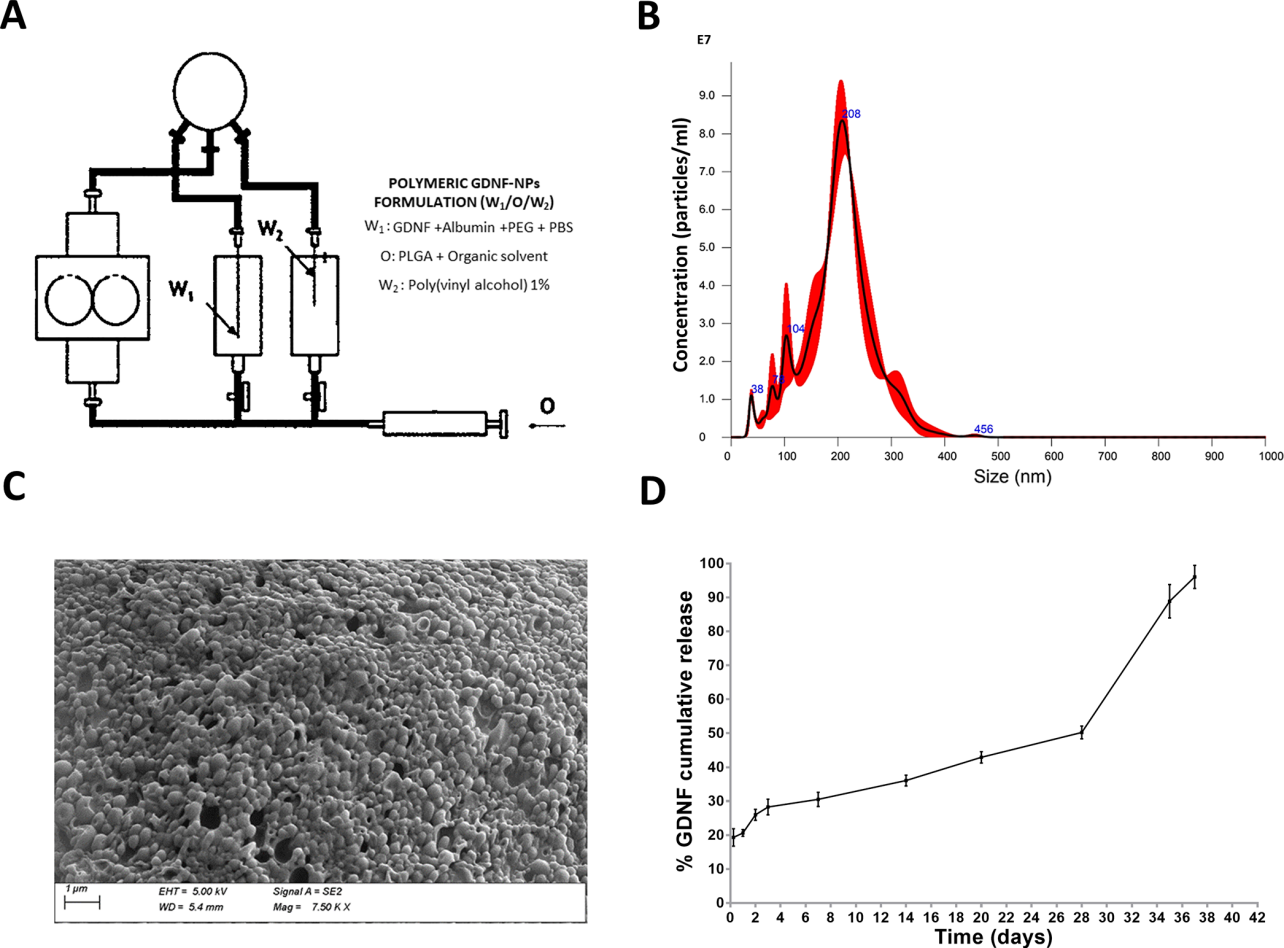
Characterization of GDNF-loaded NPs prepared using TROMS. (A) Schematic illustration of TROMS. (B) NP size determined by NTA. (C) Scanning electron microscopy images showing the morphology of freeze-dried NPs. (D) *In vitro* release profile of GDNF from NPs by determination of the supernatant protein content.

Monodisperse spherical PLGA particles were successfully obtained using the TROMS, a technology that avoids shear stress and that it is suitable for the encapsulation of labile molecules like proteins (Figure 2A).^50,51^ The mean particle size measured by NTA was 208 ± 26 nm (*n* = 4) (Figure 2B). The particle surface charge after the freeze-drying process exhibits a zeta potential of −22.8 ± 3.9 mV (*n* = 4), which represents a stable colloidal system.^52^ The encapsulation efficacy was 58 ± 8%, which corresponds to a final loading of 0.41 μg of GDNF per mg of polymer. The results were similar to those obtained for other GDNF-loaded MPs and NPs,^53,54^ but with the advantage of encapsulating GDNF under milder conditions. SEM analysis showed that GDNF-NPs were spherical in shape and confirmed that NP size distribution was uniform (Figure 2C). The release of GDNF from the NPs was biphasic, with an initial burst release produced within the first 24 h. After the initial burst produced by the release of GDNF adsorbed on the particle surface (19.10 ± 3.5%), a sustained release was observed from day 1 to day 28 (50.6%), caused by drug diffusion through the PLGA. Finally, an increase in the rate of release was observed from day 28 to 40 due to polymer degradation and 96% of the total GDNF was released within the first 40 days (Figure 2D). These results were confirmed by measuring the remaining GDNF content within NPs, showing a similar *in vitro* release profile (Figure S4, Supporting information). The percentage of residual PVA in the NPs was 8.6 ± 0.4%, which is several times lower than the values previously reported for PLGA-NPs prepared by a double emulsion method and represented a safe and acceptable quantity.^36^

### 3.3 Preparation and Characterization of the HGs (6 wt % HG and 6 wt % HG-NPs)

#### 3.3.1 HG Preparation

The HGs were formed by mixing aqueous solutions of the modified HA (HA-CD and HA-AD) (6 wt % HG) and PLGA-NPs (6 wt % HG-NPs). Importantly, NPs were noncovalently immobilized in the HA-CD solution before mixing both components with the HA-AD solution. The supramolecular assembly was instantaneous upon mixing. The incorporation of NPs resulted in a homogeneous nanocomposite-HG with a soft and rubbery consistency.

#### 3.3.2 Rheological Characterization

**Figure 3:**
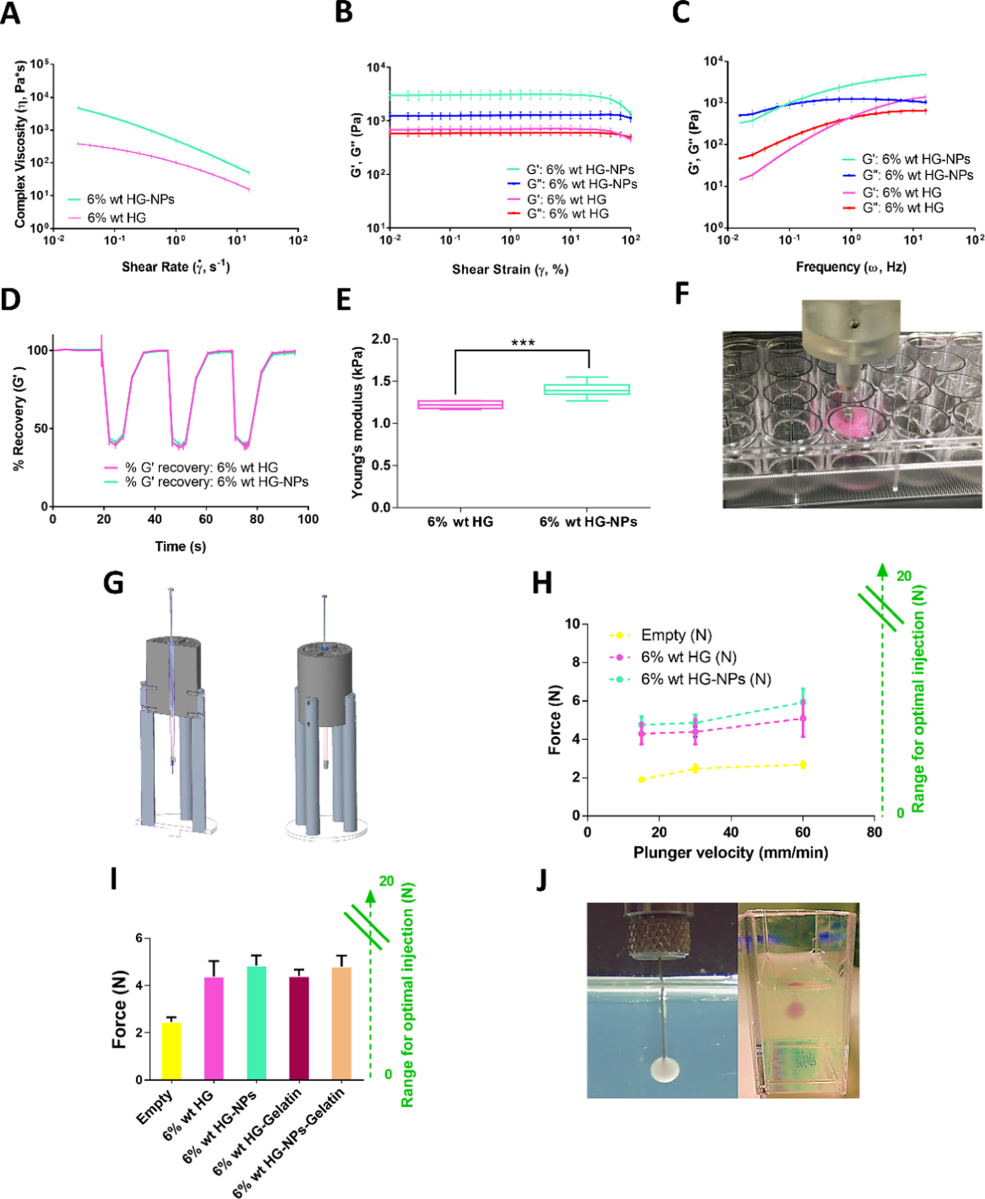
Evaluation of the rheological properties, mechanical stiffness, and injectability of the HGs. (A) Determination of complex viscosity through an oscillatory frequency sweep (from 0.15 and 100 rad s^–1^ with a shear strain of 0.5%). (B) Oscillatory strain sweep (from 0.1 to 100% and the frequency was kept at 10 s^–1^). (C) Oscillatory frequency sweep (from 0.15 and 100 rad s^–1^ with a shear strain of 0.5%). (D) Analysis of thixotropy behavior by three-step cyclic strain time sweep. (E) Determination of Young’s modulus using an indentation test. Young’s modulus was significantly increased by nanoreinforcement (***, *p* < 0.001, unpaired *t*-test). (F) Schematic of the indentation used for the determination of Young’s modulus. (G) Setup used to perform the injectability study. A cross section is shown on the left. The external aspect of the apparatus is shown on the right. (H) Determination of HG injection forces. (I) Measurement of the force required to inject HGs in air and within 4% gelatin gels at 30 mm/min. (J) Representative image of HA-HG-NPs injection on 4% gelatin gel.

Shear-thinning behavior is of greatest importance for investigating the injectability of HG for minimally invasive surgery, particularly in tissue engineering and drug delivery applications.^18,55,56^ Shear-thinning HGs show a decrease in viscosity when increasing the shear rate. Both HGs (6 wt % HG and 6 wt % HG-NPs) exhibit shear-thinning behavior, as the viscosity decreases as the shear rate increases (Figure 3A).^57^ Accordingly, this viscosity profile suggests the ability for both HGs to be injectable. Even though the viscosity increases with the addition of the NPs, it does not change the shear-thinning behavior.

We then use the storage modulus (*G*′) and loss modulus (*G*″) to analyze the HG behavior and measure its strength and calculate the linear viscoelastic range (LVER) for the subsequent analysis. First, a slight dominance of *G*′ was observed in the 6 wt % HG indicating that, at 10 Hz, the sample behaves more like viscoelastic solid (*G*′ > *G*″) than viscoelastic liquid (Figure 3B). The combination of NPs and HG provided a stiffer structure than empty HGs, which is demonstrated by a 4-fold increase in the *G*′ value. This effect was previously demonstrated by other studies where HA-HGs were combined with PLGA.^58,59^ Moreover, the NPs made the HG increase the solid-like character as the ratio *G*′/*G*″ increased significantly (*G*′ ≫ *G*″). Both materials showed a stable constant behavior for almost all of the shear strain range analyzed, to exhibit at the end a drop in the storage and loss moduli. The yield point occurs on the empty HG at a lower strain than the NP-filled HG, which is consistent with the previous viscosity results. Overall, these results indicate that spherical PLGA-NPs establish strong physical interactions with the HA network, as they are active crosslinkers of the HG.^30−32^ These interactions between the NPs and the HA network may be due to hydrophobic interactions between the PLGA of the NPs and the AD, both hydrophobic molecules. Also, favorable energetic adsorption of the NPs to the HA network may cause this crosslinking effect. In the end, both the guest–host interactions and the NPs help to provide a mechanically compatible solid-like material.^60−63^

Next, the behavior of the synthesized HG was investigated at both high and low frequencies using a frequency sweep test.^38^ This analysis shows how both formulations transition from liquid behavior to solid behavior, when increasing the frequency, with positive slopes on the *G*′ and *G*″ (Figure 3C). Both profiles of this graph resemble a Maxwell behavior for untangled polymers, where the crossover frequency is an indicator of the reversible crosslinking characteristic of the HG. Interestingly, the addition of NPs, noncovalently immobilized, causes the crossover frequency to decrease on the loaded HG, and thus the relaxation time (λ) increases. Therefore, the addition of the NPs causes a higher opposition to flow compared to the plain HG. Still, both compositions were easily injectable as demonstrated in the following sections.

To evaluate the self-healing properties of the HGs, a three-step oscillation method was performed. The ability of the HGs to recover their mechanical properties was investigated by studying the behavior of *G*′ at high and low strain values. The results were expressed as a percentage of *G*′ recovery (Figure 3D). The recovery test showed that both HGs showed a fast decrease in *G*′ at high strain values, which were instantly recovered after cessation of the high shear and the application of low strain values again. This confirms the potential of the developed HGs to recover their mechanical strength upon injection.^64^

The biomaterial environment and mechanical properties have great relevance for the clinical success of cell therapies. Therefore, the mechanical properties of the material should be similar to those of brain tissue. In this sense, the brain is a soft tissue, with Young’s modulus between 1 and 14 kPa in humans, and biomaterials with stiffness 0.1–20 kPa are preferred to support neuronal cell proliferation.^24,65^ The stiffness of both HGs was analyzed by measuring Young’s modulus from indentation tests (Figure 3E,F). The developed HGs showed Young’s moduli of 1,22 ± 0.04 kPa (6 wt %) and 1.39 ± 0.08 kPa (6 wt % HG-NPs). These values suggest potential compatibility with brain tissue and a suitable stiffness for neural differentiation.^24,65^ A higher Young’s modulus was observed when the NPs were incorporated into the HG, which demonstrates the relevance of HG reinforcement with nanospheres as an effective strategy to modulate the matrix stiffness for the design of biomaterials for tissue engineering (Figure 3E).^63^

Although the study of storage modulus and loss modulus and the analysis of the shear-tinning and self-healing behavior are suitable predictors of biomaterial injectability, the injection force is the most important parameter to conclude when a material is clinically valid for injection.^38^ Here, we quantitatively determined the injectability of the prepared HGs through a Hamilton syringe with a 27G needle. The measured injection force would correspond to the force that the end-user has to exert on the syringe plunger top for extracting the HG from the syringe in a real situation. This is not a material parameter but is a combination of the viscoelastic properties of the material, size of the needle, and the syringe used.^66^Figure 3G is a schematic of the manufactured holder used. On the left, the external aspect of the holder is shown. The central area shows both the barrel at the bottom and the plunger at the top. The external aspect of the holder is displayed on the right-hand side of Figure 3G.

The force to move the plunger was higher as the injection flow rate increased (Figure 3H). In addition, there was an increase in the force required to inject the nanoreinforced material compared to the injection of the material without NPs. At a flow rate of 15 mm/min, the injection force required for a 6 wt % HG was close to 4.3 N while the injection force required for a 6 wt % HG-NPs was close to 4.8 N, representing an increment of 10%. This difference was increased progressively to 16% for the fastest case studied (60 mm/min) (Figure 3H).

The higher viscosity, storage and loss moduli, and the increment of the stiffness observed for the 6 wt % HG-NPs in the previous analyses involved a higher force to inject the biomaterial. In all cases, the forces were within the valid range for a clinical injection. Thus, materials with injection forces < 20 N are potentially injectable at the clinical level.^38^ The highest force recorded in our study did not exceed 7 N, which indicates that the developed biomaterial is clinically injectable (Figure 3H).

The HGs not only demonstrated good injectability when injected into the air but also when injected into a gelatin gel at 4%, which simulates the mechanical properties of the brain.^38,67^ Thus, the HG injections into the gelatin gel did not increase the force required for its administration, which confirms the excellent injection capability of the developed system (Figure 3I,J).

Overall, the rheological characterization confirms that the addition of the NPs does not change the general behavior of the HG, even though it makes the HG stiffer. The injectability of this material does not change significantly due to the NPs. Therefore, the combination of HG and NPs provides an effective strategy to deliver at local site the desired treatment.

#### 3.3.3 Morphological Analysis of the Multifunctional HGs by SEM

**Figure 4:**
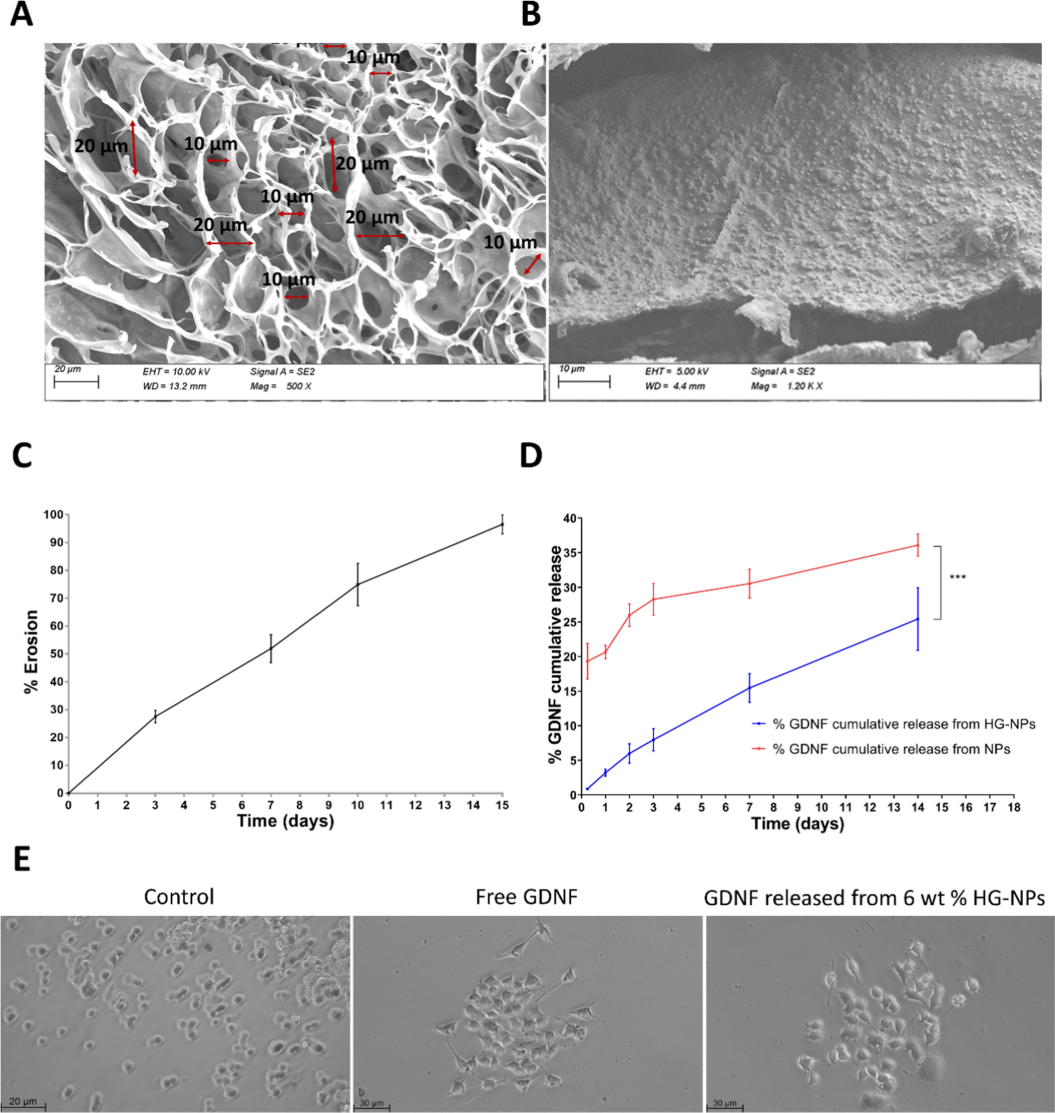
Morphological characterization and in vitro evaluation of 6 wt % HG-NPs. (A, B) SEM analysis of freeze-dried HGs. The HGs showed pores with a size in the range of 10–20 μm. (A) and (B) NPs appear physically embedded into the HG network. (C) In vitro erosion of the 6 wt % HG-NPs over 15 days. (D) Comparative cumulative release profile of GDNF: NPs *vs* 6 wt % HG-NPs. The incorporation of NPs into the HG produced a significant reduction of drug release at 2 weeks (***, *p* < 0.001, paired *t*-test). (E) Analysis of GDNF activity in vitro. Representative images of control PC12 cells treated with free GDNF or with GDNF released from 6 wt % HG-NPs (magnification: 20×).

Next, the morphological properties of the 6 wt % HG and 6 wt % HG-NPs were analyzed by SEM, which reveals the porosity and nature of the HG structure. All of the developed HGs had a homogeneous surface and a porous interior structure. The pore size of all HGs was in the range of 10–20 μm (Figure 4A). The NPs were physically embedded and uniformly dispersed into the HG network in the nanoreinforced HG (Figure 4B). The morphological analysis suggests a certain NP absorption in the HG, which would induce higher friction between components. Therefore, these physical interactions could be responsible for the increment of all studied parameters (viscosity, storage modulus, loss modulus, and injection forces).^68,69^ These results are consistent with previous studies, where polymeric NPs have been successfully incorporated within the HG network to increase the cross-linked density and to obtain reinforced nanocomposite HGs.^18,60,70^

#### 3.3.4 Degradation Test of Hybrid Systems (6 wt % HG-NPs)

The HG degradation influences the release rate of biomolecules from the hybrid system and therefore, it is an important parameter to provide a controlled GDNF release.^71^ The 6 wt % HG-NPs slowly degraded, showing a partial degradation of 42% after one week (Figure S5, Supporting information). Accordingly, the carbazole assay demonstrated a linear rate of erosion over 14 days (Figure 4C), which supports the idea that our drug delivery platform could influence drug release kinetics over a few weeks through its linear degradation.^72^

#### 3.3.5 *In Vitro* Release Study of GDNF from Hybrid Systems (6 wt % HG-NPs)

Despite the proven potential of GDNF as a treatment for PD, its clinical application has been hampered by safety and efficacy issues associated with GDNF’s short *in vivo* half-life.^50^ As an alternative, hybrid systems such as nanoreinforced supramolecular HGs may overcome this issue by providing protein protection from degradation and sustained drug release over time.^73^ The release of GDNF from the 6 wt % HG-NPs occurred in a sustained manner and the cumulative release rate reached 25.4 ± 4.5% in 2 weeks. The incorporation of NPs into the supramolecular HG allowed a more sustained release profile, as well as a significant reduction of drug release at two weeks (25 *vs* 36%) (Figure 4D). The drug release measured for these two materials has been fitted to the Ritger–Peppas power law model (eq 1). For the NPs system, the obtained parameters have been *k* = 22.8 and *n* = 0.162. For the case of the HG-NPs, they were *k* = 3.04 and *n* = 0.83. Remarkably, the incorporation of NPs into the HG considerably reduced the initial burst release from PLGA-NPs. Initially, this release profile may be attributed to the diffusion through the PLGA-NPs and the HG matrix. This effect could be because the HG forms a cross-linked and porous matrix through the proteins that have to diffuse.^58^ However, the further erosion of the HG in the second part of the study reduced the differences between the release of GDNF from the HG with respect to the GDNF contained in the NPs. Given that the degradation of PLGA is not very significant during this period, the GDNF can be released mostly by diffusion from areas near the surface of NPs. However, it is possible that the presence of the surrounding HA-HG has a delayed or deposited effect on the release profile of the GDNF during this phase. Similar results were previously reported with vascular endothelial growth factor (VEGF) and brain-derived neurotrophic factor (BDNF)-MPs embedded in HA-HG.^58^ This effect could be because the HG forms a cross-linked and porous matrix through the proteins that have to diffuse.^58^ Thus, the nanoreinforcement proved an effective strategy to improve not only the HG robustness but also to achieve a controlled drug release from the HG.

#### 3.3.6 *In Vitro* Bioactivity Assay of GDNF from Hybrid Systems (6 wt % HG-NPs)

The successful development of a strategy based on GDNF-NPs embedded within an HA-HG requires the preservation of the biological activity of the neurotrophic factor through all of the manufacturing processes. Therefore, the bioactivity of GDNF released from the 6 wt % HG-NPs was next evaluated using a PC12 neurite outgrowth assay. PC12 cells, which possess the GFRα1 and RET receptors, change their phenotype and develop neurites after treatment with biologically active GDNF, which is visualized by the sprouting of neurites.^74,75^ After 10 days of treatment with GDNF released from the 6 wt % HG-NPs, PC12 cells acquired a phenotype associated with sympathetic neuron-like cells, which includes the inhibition of proliferation and the outgrowth of neurites (Figure 4E). On counting the neurite outgrowth, no significant differences were found between cells treated with free GDNF (11 ± 3.46 neurites) and those treated with GDNF released from HG-NPs (11.5 ± 4.04 neurites) (*p* > 0.05, unpaired *t*-test), indicating that GDNF remains bioactive after being released from the HG-NPs. Collectively, these results demonstrate that the neurotrophic factor remains bioactive after its encapsulation and release from the drug delivery platform.

#### 3.3.7 Cell Viability and Neural Biocompatibility

HA is the major component of the brain ECM and has a valuable role in neural homeostasis. Precisely, HA influences different cell activities such as cell migration, proliferation, and differentiation, among others.^76^ Nonetheless, due to the introduction of different modifications in the HA and the combination with PLGA-NPs, cell viability and neural biocompatibility were assessed.

**Figure 5:**
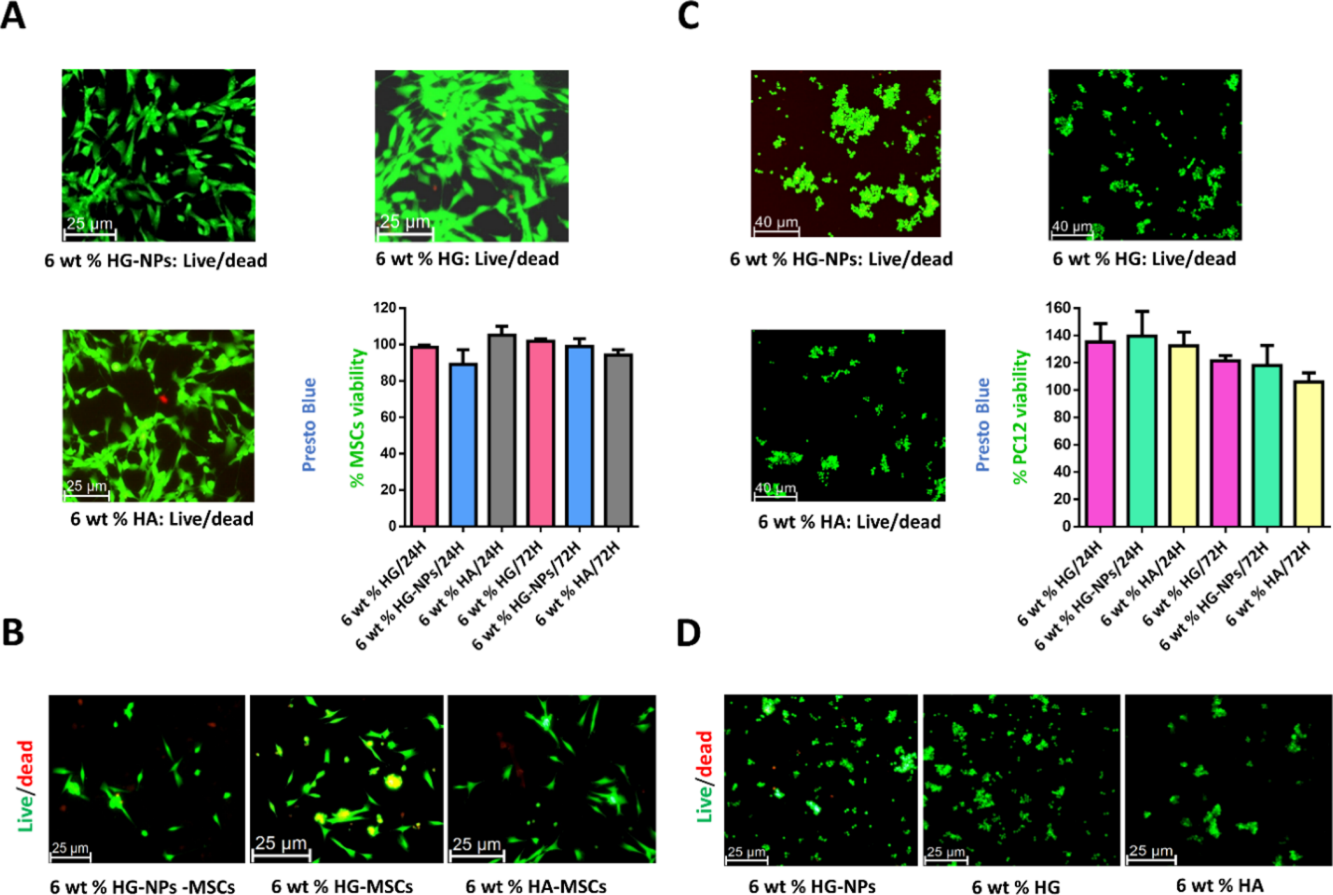
*In vitro* cell viability and neural biocompatibility. (A) Live/dead and PrestoBlue assay of MSCs cells treated with extracts according to ISO 10993-5 for *in vitro* cytotoxicity. (B) Live/dead assay of encapsulated MSCs within HGs. (C) Live/dead and PrestoBlue assay of PC12 cells treated with extracts according to the ISO 10993-5 for *in vitro* cytotoxicity. (D) Live/dead assay of encapsulated PC12 within HGs.

From a cell compatibility standpoint, PrestoBlue and Live/dead test revealed that survival rates for modified and nanoreinforced HGs were higher than 90% compared to extracts of unmodified HA (Figure 5A). For encapsulated cells, Live/dead measurements showed a similar trend without differences in cellular morphology between groups (Figure 5B). These results confirm that the modifications performed in the HA and the incorporation of the NPs did not have a toxic character, and therefore, the novel HGs would be safe.

Further, the neural compatibility of the developed nanoreinforced HG was also studied in PC12 cells, a widely characterized neurotoxicology model.^77,78^ A notable increase in cell proliferation was observed in PC12 treated with the HG extracts at 24 and 72 h (Figure 5C). The highest proliferation was observed at 24 h for 6 wt % HG-NP samples. Importantly, at 72 h, 6 wt % HG and 6 wt % HG-NPs extracts produced higher proliferation than the control sample of HA extract. The cells directly exposed to the different HGs did not show significant differences in morphology compared to control samples of HA and most of the PC12 cells were alive, indicating good cell compatibility (Figure 5D). These results are in good agreement with previous observations reported on HA-HGs combined with biodegradable NPs, where good biocompatibility with human umbilical cord endothelial cells was also demonstrated.^18^

### 3.4 3D Environment Provided by the Nanostructured HG Changes MSC Transcriptomics and Boosts Their Therapeutic Effects

**Figure 6:**
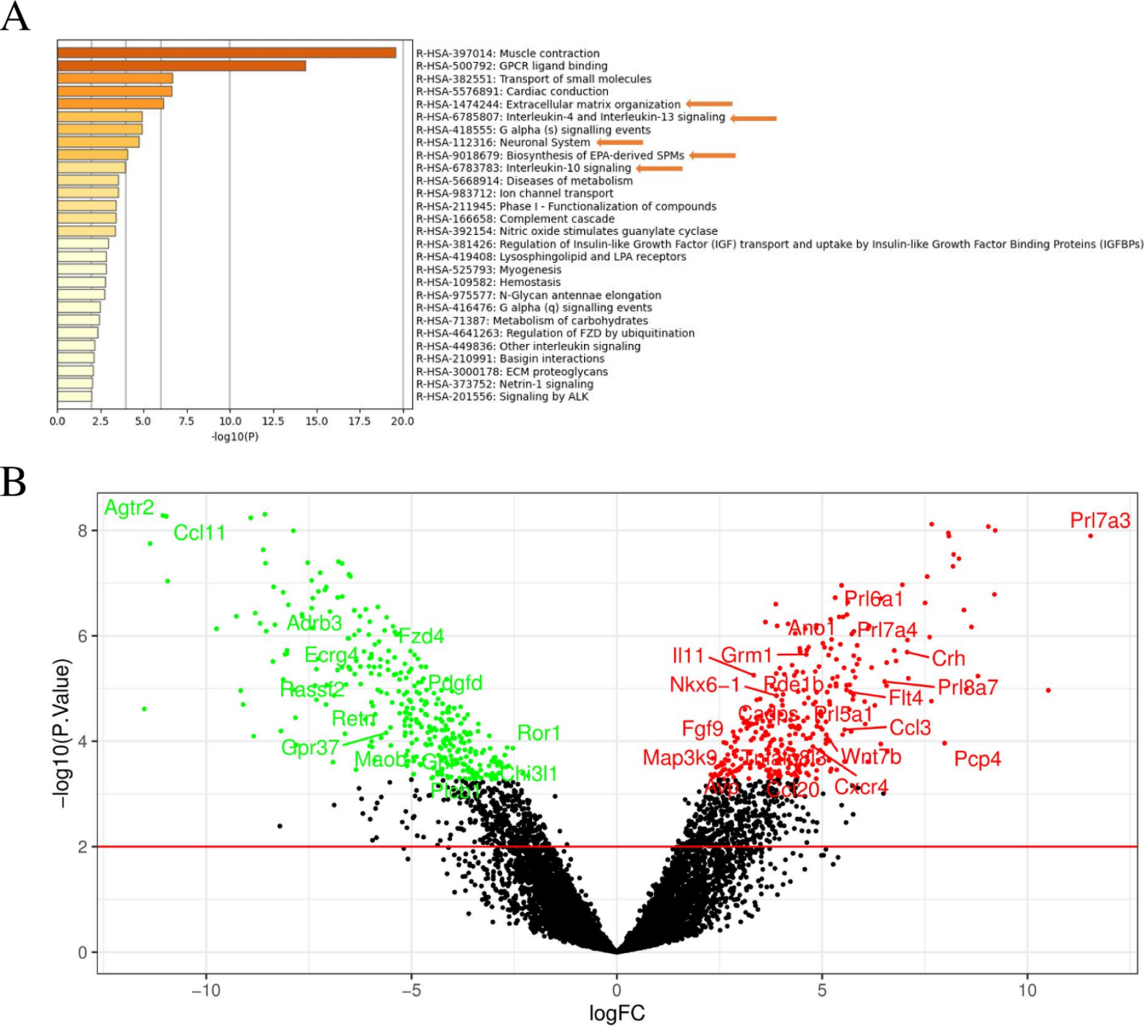
(A) Functional enrichment analysis by Metascape. Bar chart of clustered enrichment ontology categories. Reactome database using default settings (minimum overlap: 3; minimum enrichment: 1.5; *p* < 0.01). (B) Volcano plot displaying representative differentially expressed genes in RNA-seq data between nanoreinforced HG-included and nonincluded MSCs. The red dots represent the upregulated expressed transcripts. Green dots represent the transcripts whose expression is downregulated. Positive *x*-values represent upregulation, and negative *x*-values represent downregulation.

The administration of stem cells within HGs has emerged as a powerful strategy to enhance graft survival and integration and boost their therapeutic efficacy.^11,23^ Moreover, although it is known that the 3D environment and the interaction of cells with ECM components change stem cell transcriptomics and impact stem cell viability, function, and fate, the mechanisms underlying these events remain poorly understood. Therefore, the transcriptomic effect of culturing MSCs within the nanoreinforced HG was studied through pathway analyses and gene expression changes, to elucidate the molecular mechanisms in which the differentially expressed genes are involved. One of the known characteristics of MSCs is their immunomodulatory capacity through the release of anti-inflammatory cytokines and inhibition of pro-inflammatory ones.^79^ This fact becomes relevant in a context where the death of cell implants is partly due to the action of the immune system and where there is widespread neuroinflammation as in PD.^4,23^ This neuroinflammation is mainly caused by microglia, the resident macrophages of the CNS.^4^ In PD, these cells seem to be overactivated and might be responsible for graft rejection and low-rate survival.^8,80^ As shown in Figure 6A, interleukin (Il)-4 and Il-13 signaling (log *P*: −4.90), as well as Il-10 signaling (log *P*: −3.93) were significantly enriched in MSCs embedded within the nanostructure HG compared to those cultured in 2D (Table S1, Supporting Information), suggesting that the HG enhances the proven anti-inflammatory effects of MSCs. In addition, an upregulation of the *Il-11* gene expression, another anti-inflammatory cytokine, was observed (Figures 6B and S6, Supporting Information). Also, the biosynthesis of eicosapentaenoic acid (EPA)-derived specialized pro-resolving mediators (SPMs) was enriched (log *P*: −4.07). These ω-3 polyunsaturated fatty acid (PUFA)-derived SPMs present anti-inflammatory and pro-resolving effects.^81,82^ Even more, enrichment in the expression of *Socs2*, encoding for a protein member of the suppressor of cytokine signaling family was found. This anti-inflammatory effect enhanced by the biomaterial may be of great interest not only in preventing graft rejection by the immune system but also in treating the underlying neuroinflammation process of PD and can contribute to delay the progressive course of the disease. Moreover, this anti-inflammatory effect could be of great value in designing new cell therapy strategies for PD. For instance, mixed striatal grafts of MSCs with dopaminergic progenitors or induced pluripotent stem cell-derived dopaminergic neurons might increase cell survival by reducing α-synuclein aggregation into grafted dopaminergic cells. In addition, this approach could serve to better understand *in vivo* the interaction between inflammation and α-synuclein aggregation.

Due to the 3D environment, the ECM organization pathway (log *P*: −6.12) presented several differentially expressed genes related to its remodeling, mainly matrix metalloproteinases (MMPs), a disintegrin and MMP with thrombospondin motifs (ADAMs) family members. Enrichment of ECM-related functional groups is positive, as cell-ECM interactions significantly improve cell implant survival. In fact, the lack of this interaction promotes cell-programmed death, especially in stem cells.^83^

Regarding stem cell differentiation, multiple authors have reported that MSCs can differentiate into neuron-like cells under certain conditions, such as 3D environment and composition.^84,85^ Given that the nanoreinforced HG mimics the brain’s ECM and meets similar elastic modulus and stiffness of brain tissue, several genes related to the neuronal system are differentially expressed (log *P*: −4.72), such as *Nrxn3*, *Slc6a*12, *Grm1* or different potassium voltage-gated channels. This result reinforces the hypothesis that MSCs could differentiate into the neuronal lineage, suggesting that these cells may have the machinery needed to differentiate into neuron-like cells under certain conditions. Other genes usually expressed in neurons or brain-resident cell types present significant changes in their expression, such as *Mao-B* or *Ret*, a GDNF co-receptor. Related to GDNF effects on MSC grafts included in HGs, this overexpression of Ret may increase its efficacy/biological effect. In addition, GDNF promotes cell integration by upregulating the expression of genes of the MAPK pathway as previously shown.^86^ Finally, the HG loaded with cells and the HG loaded with empty NPs and cells were compared to assess the possible effect of the NPs on the cells, either by changing the mechanical properties of the HG or by the composition of the NPs (PLGA). In this case, no differences were observed between the groups, indicating that HG stiffening does not alter the transcriptome of the embedded cells (Table S2, Supporting information).

In conclusion, we have provided a better understanding of how the 3D environment provided by the nanostructured HG impact the functions of the encapsulated cells that may help in the design of brain tissue engineering strategies for PD.

## 4 Conclusions

A nanoreinforced HG for the dual administration of GDNF and MSCs was successfully prepared and characterized. The use of cells, neurotrophic factors, and biomaterials in a single therapeutic strategy could have enormous potential in the treatment of neurodegenerative diseases such as PD, where a progressive loss of dopaminergic neurons occurs. First, the incorporation of NPs in the supramolecular guest–host HG enhanced the mechanical properties of the biomaterial compared to conventional HA gels, acting as physical crosslinkers of the HG. Then, we demonstrated that the developed HG enhances the anti-inflammatory properties of MSCs, boosts their relation with the ECM, and promotes the differentiation toward neuron-like cells. In summary, the suitable strength, excellent self-healing properties, good biocompatibility, and ability to enhance MSCs regenerative potential make this nanoreinforced HG a good candidate for drug and cell administration to the brain.

## Supporting Information Available

^1^H NMR spectra of HA-CD and HA-AD in D_2_O, HA-AD with dimethylsulfone in D_2_O to quantify the residual amount of TEA, cumulative release of GDNF after NPs disruption with DMSO, % residual weight (RW) of 6% wt HG-NPs over seven days, heat map of differentially regulated genes due to the 3D environment, functional analysis of the HG-NP-MSCs *vs* MSCs (Table S1), and LIMMA output of the whole RNA-seq experiment (Table S2) (ZIP)
