# Supplementary material for: Encapsulation of MSCs and GDNF in an Injectable Nanoreinforced Supramolecular Hydrogel for Brain Tissue Engineering
Source: Biomacromolecules. 2022 Oct 26;23(11):4629–44. doi: 10.1021/acs.biomac.2c00853 (PMC9667499; doi:10.1021/acs.biomac.2c00853)
Supplement: Supplementary file 1 — bm2c00853_si_001.zip [file bm2c00853_si_001.zip › P_Torres_et_al_Supporting_Information.docx]

Supporting Information

Encapsulation of MSCs and GDNF in an injectable nanoreinforced supramolecular hydrogel for brain tissue engineering

Pablo Vicente Torres-Ortega ^(1,2, †)^, Rubén Del Campo-Montoya ^(1,2, †)^, Daniel Plano ^(1,2)^, Jacobo Paredes ^(3)^, Javier Aldazabal ^(3)^, María-Rosario Luquin ^(2,4)^, Enrique Santamaría ^(5)^, Carmen Sanmartin ^(1,2)^, María J. Blanco-Prieto ^(1,2)^ ^§*^, Elisa Garbayo ^(1,2)^ ^§*^

^1^ Department of Pharmaceutical Technology and Chemistry, Faculty of Pharmacy and Nutrition, University of Navarra, C/ Irunlarrea 1, 31008 Pamplona, Spain

^2^ Navarra Institute for Health Research, IdiSNA, C/ Irunlarrea 3, 31008 Pamplona, Spain

^3^ Tecnun, School of Engineering, University of Navarra, C/ Manuel de Lardizábal 15, 20018 San Sebastián, Spain

^4^ Department of Neurology and Neurosciences, Clínica Universidad de Navarra, Pamplona, C/ Pío XII 36, 31008 Pamplona, Spain.

^5^ Clinical Neuroproteomics Unit, Navarrabiomed, Hospital Universitario de Navarra (HUN), Universidad Pública de Navarra (UPNA), Instituto de Investigación Sanitaria de Navarra (IdisNa), Pamplona, Spain.

† Authors contributed equally.

§ M.J. Blanco-Prieto and E. Garbayo are equal senior authors

*Corresponding authors at: Department of Pharmaceutical Technology and Chemistry, Faculty of Pharmacy and Nutrition, Universidad de Navarra, C/Irunlarrea 1, 31008 Pamplona, Spain.

E-mail addresses: egarbayo@unav.es (E. Garbayo), mjblanco@unav.es (M.J. Blanco-Prieto).

Supporting Information: ^1^H NMR spectra of HA-CD (Figure S1) and HA-AD (Figure S2) in D_2_O, HA-AD with dimethylsulfone in D_2_O to quantify the residual amount of TEA (Figure S3), cumulative release of GDNF after NPs disruption with DMSO (Figure S4), % Residual weight (RW) of 6% wt HG-NPs over seven days (Figure S5), heat map of differentially regulated genes due to the 3D environment (Figure S6), functional analysis of the HG-NPs-MSCs vs. MSCs (Table S1) and LIMMA output of the whole RNA-seq experiment (Table S2).


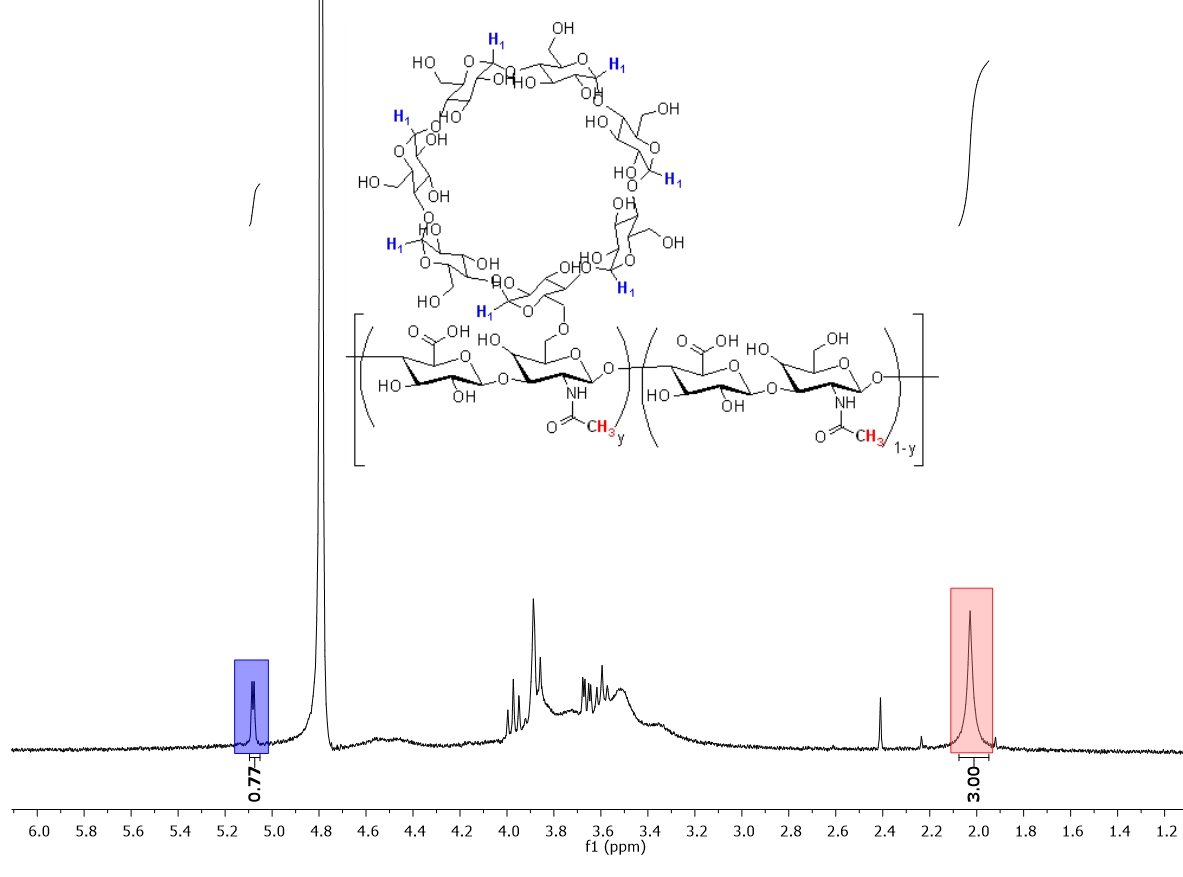


Figure S1. ^1^H NMR spectra of hyaluronic acid (HA) modified with cyclodextrin (CD) in D_2_O. Modification of HA with pendant CD (10.6 ± 1.5 %) was determined by integration of the signal for the hydrogen on position 1 of CD (7 Hs, shaded blue) relative to the signal for *N*-acetyl singlet of HA (3Hs, shaded red).


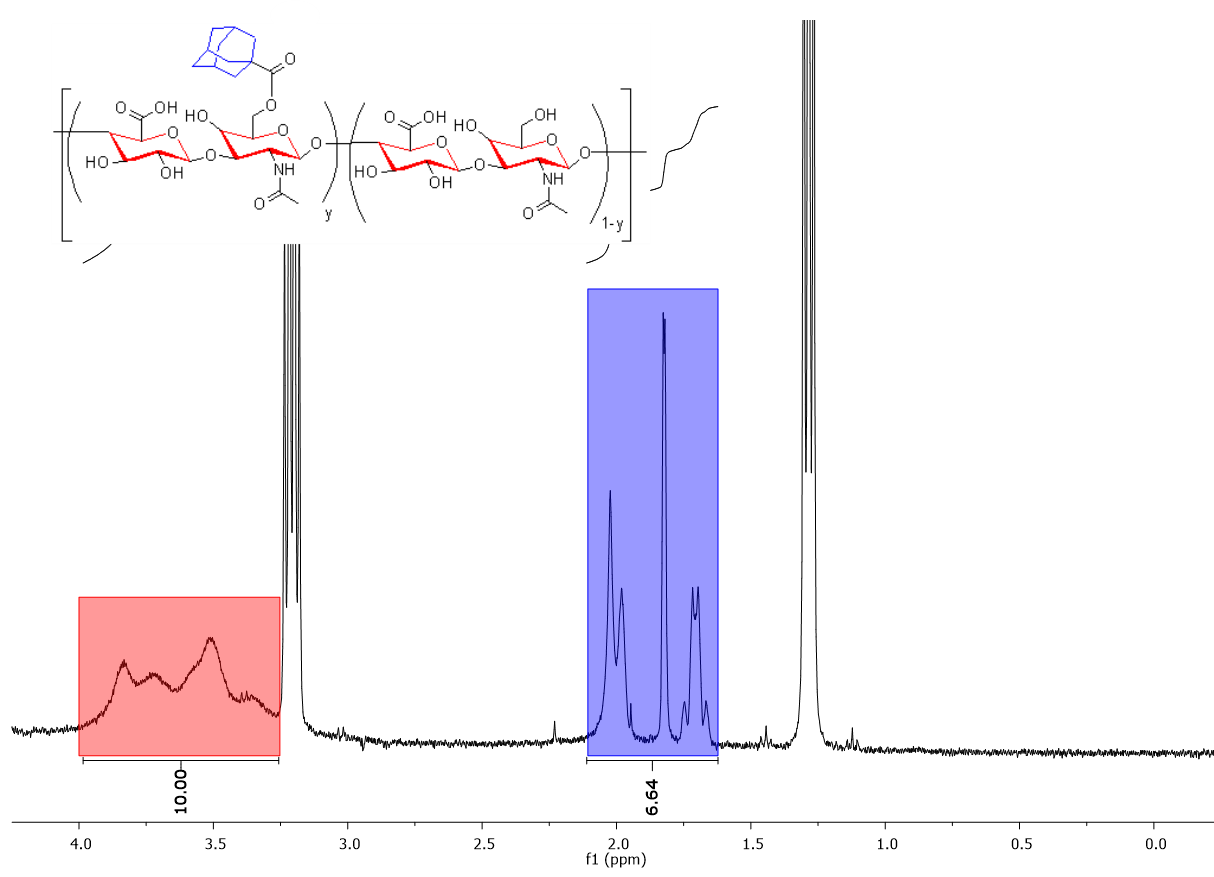


Figure S2. ^1^H NMR spectra of HA modified with adamantane (AD) in D_2_O. Modification of HA (40.3 ± 3.5 %) with pendant AD was determined by integration of the adamantane hydrogens (15Hs, shaded blue) relative to the sugar ring of HA (10Hs, shaded red).


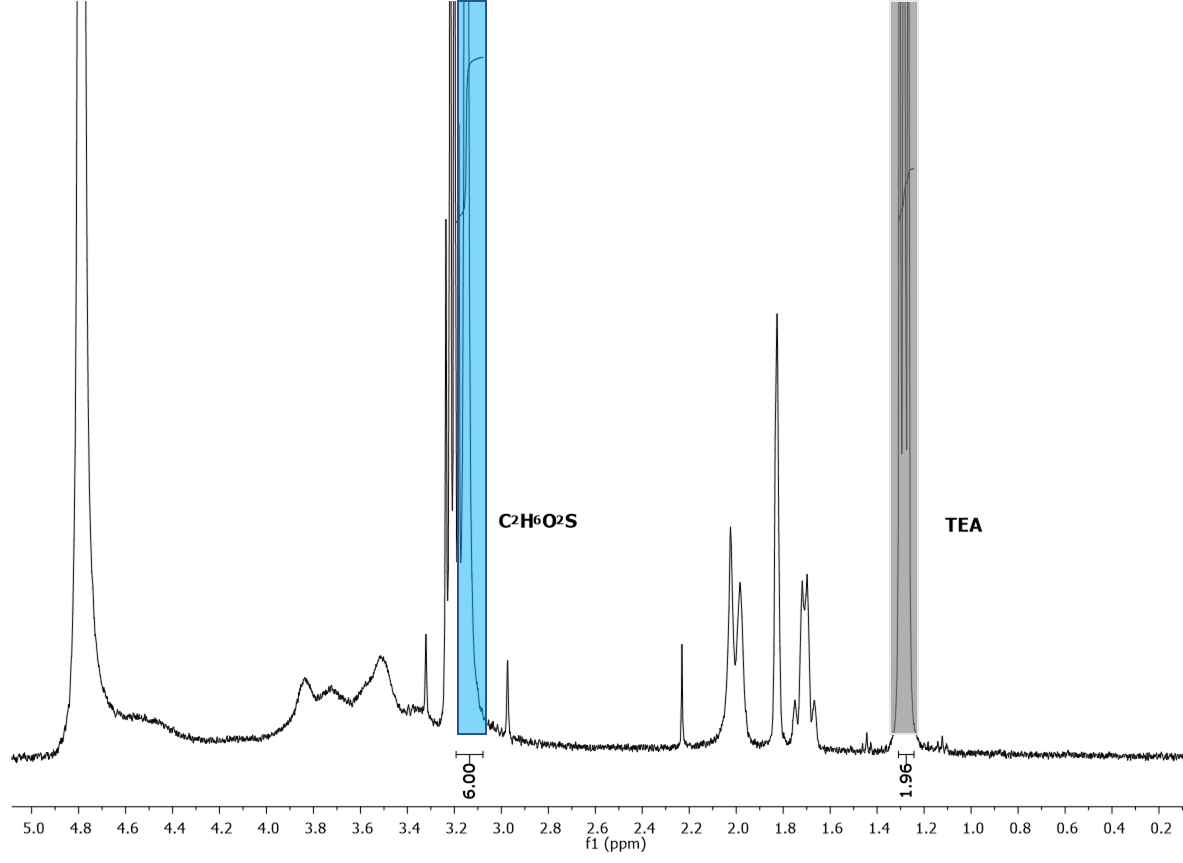


Figure S3. ^1^H NMR spectra of HA-AD with dimethylsulfone in D_2_O. The residual amount of Triethylamine (TEA) resulting from the HA-AD synthesis was determined by integration of the methyl triplet of TEA (9Hs, shaded grey) relative to the singlet of dimethylsulfone (6Hs, shaded blue).


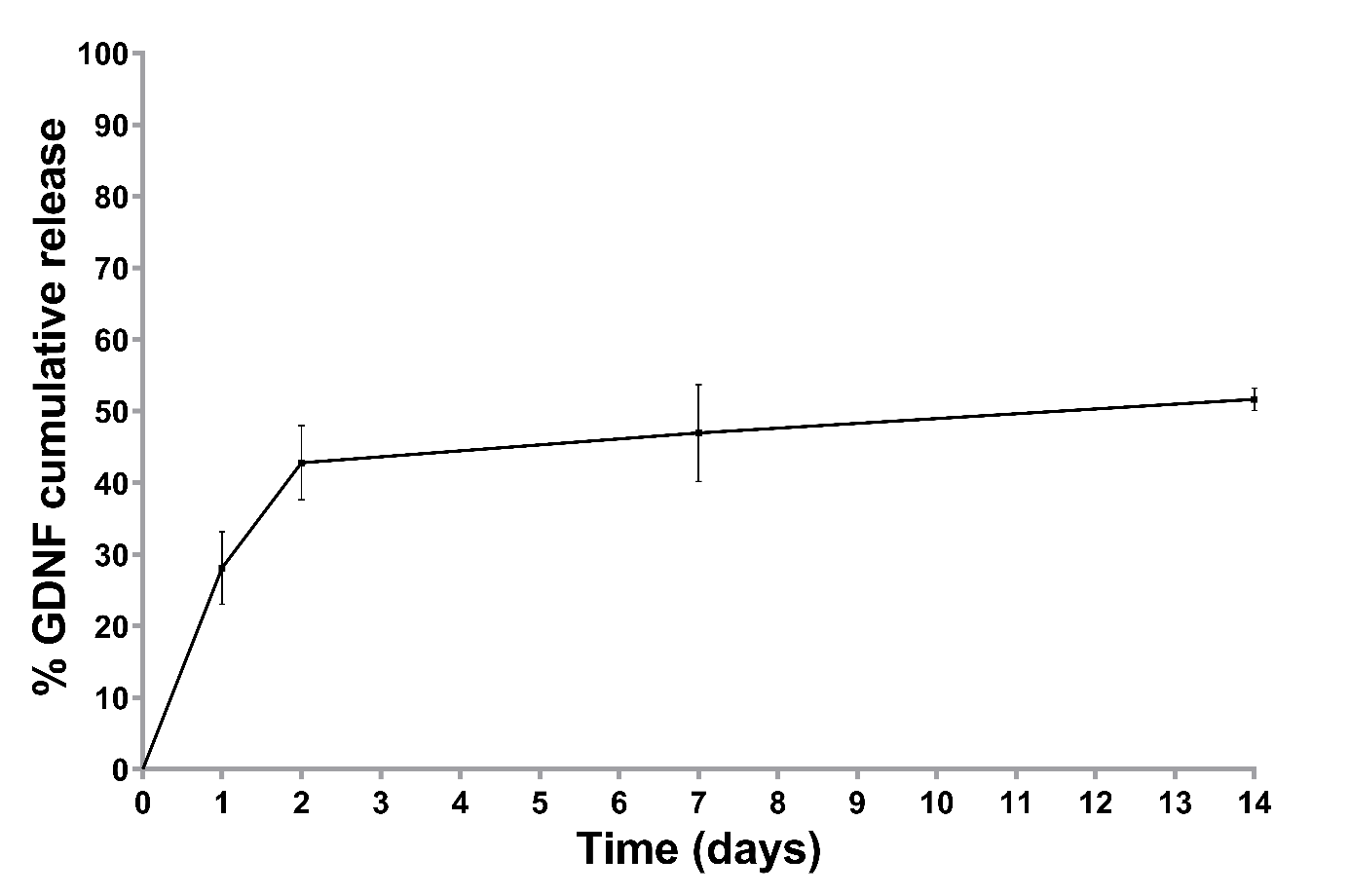


Figure S4. Cumulative release of GDNF after NPs disruption with DMSO.


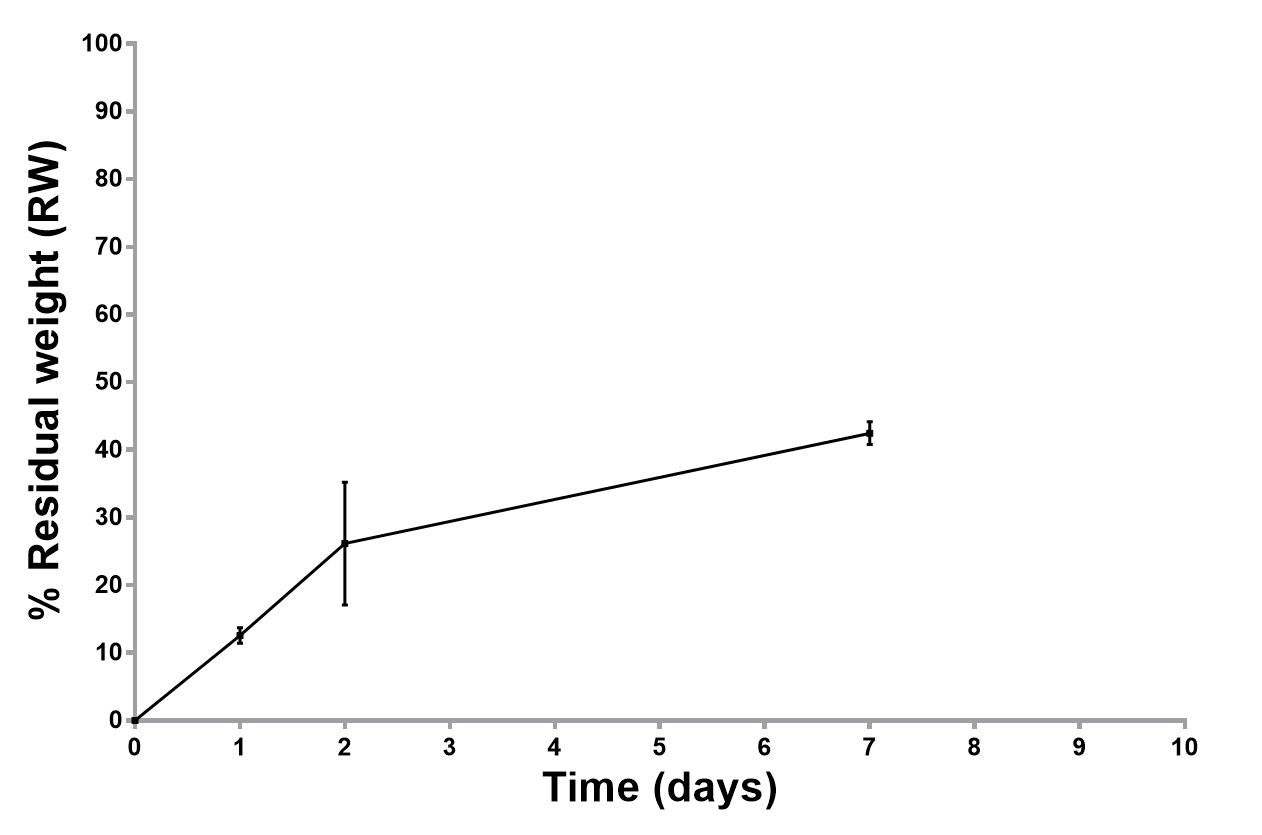


Figure S5. % Residual weight (RW) of 6% wt HG-NPs over seven days.


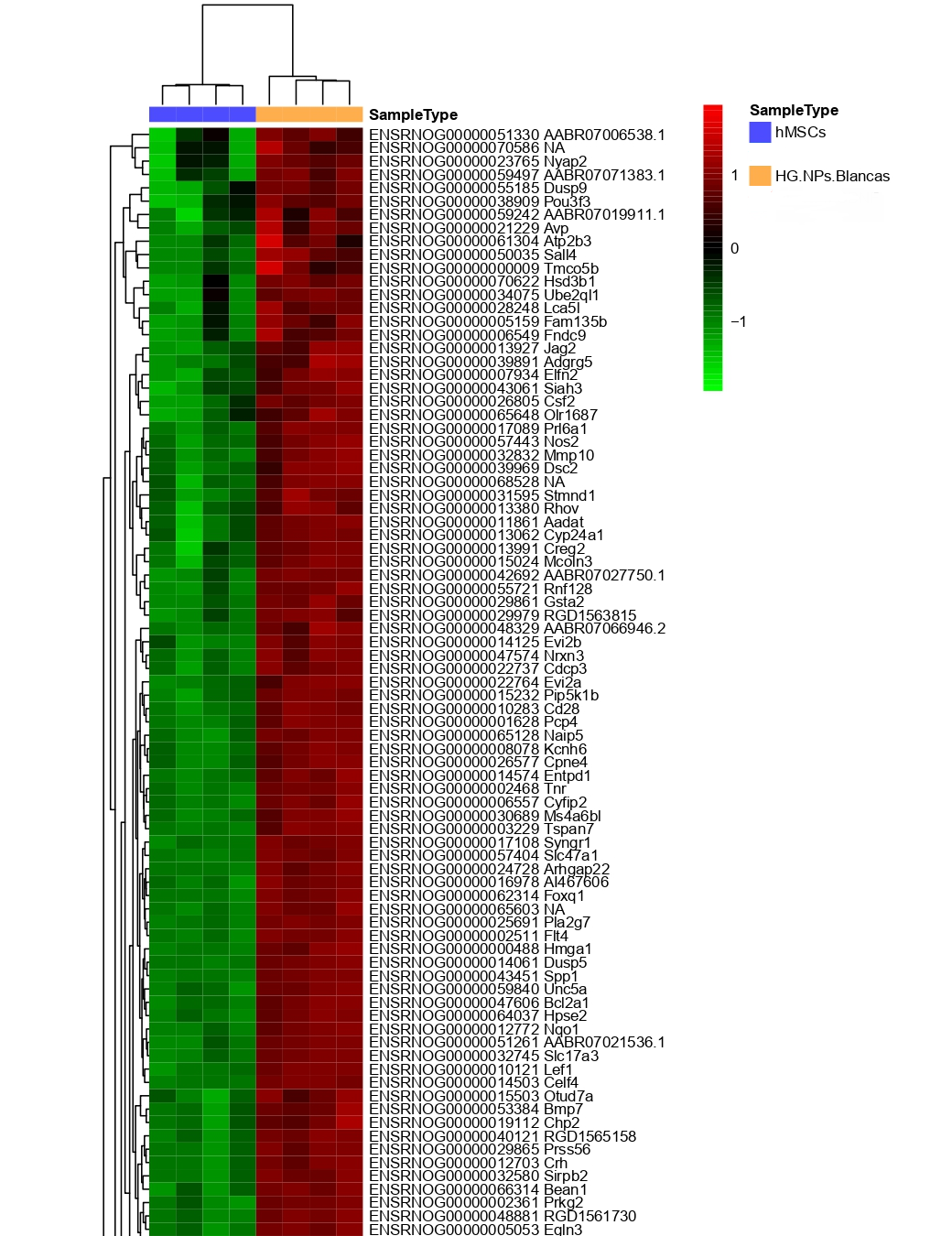

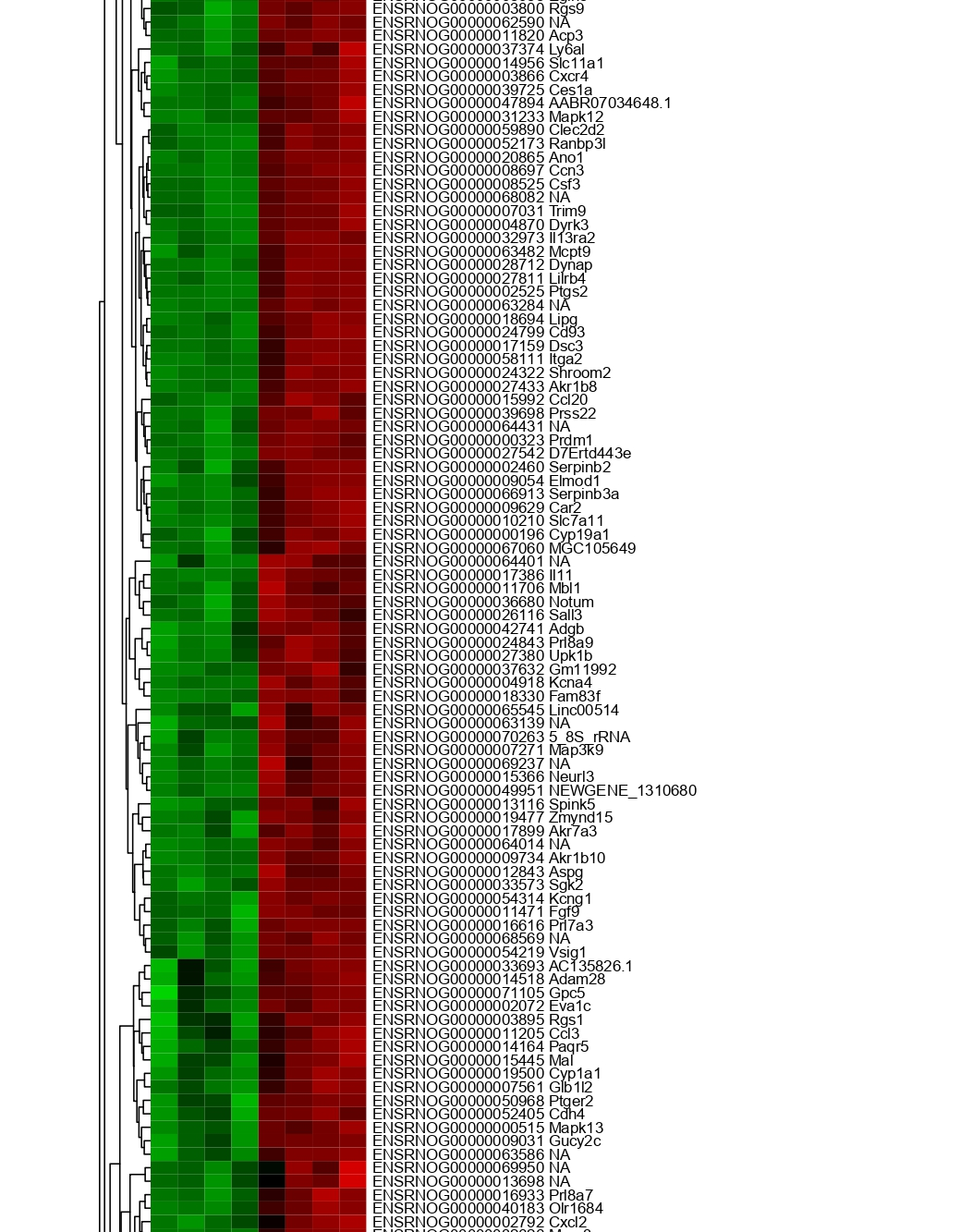

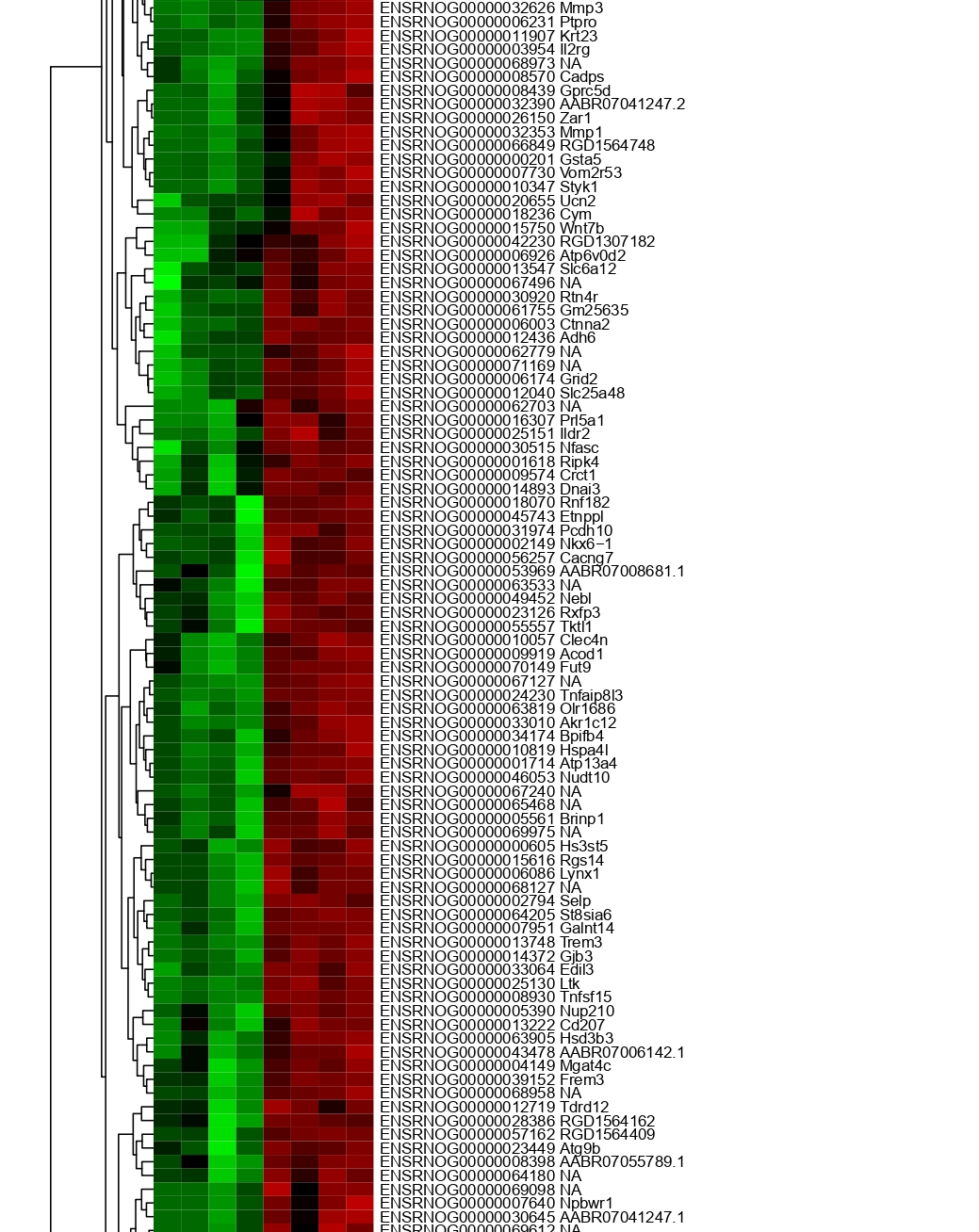

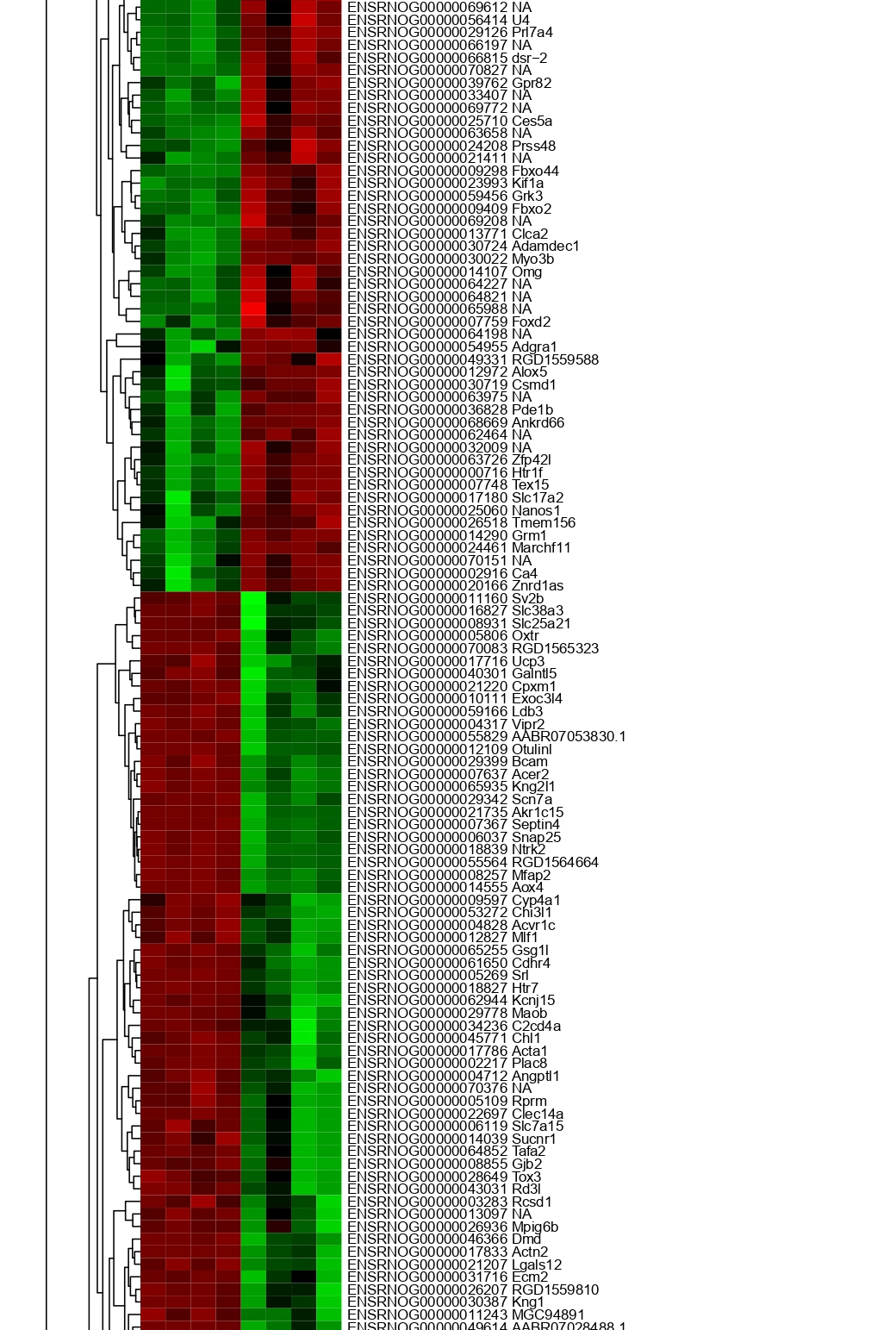

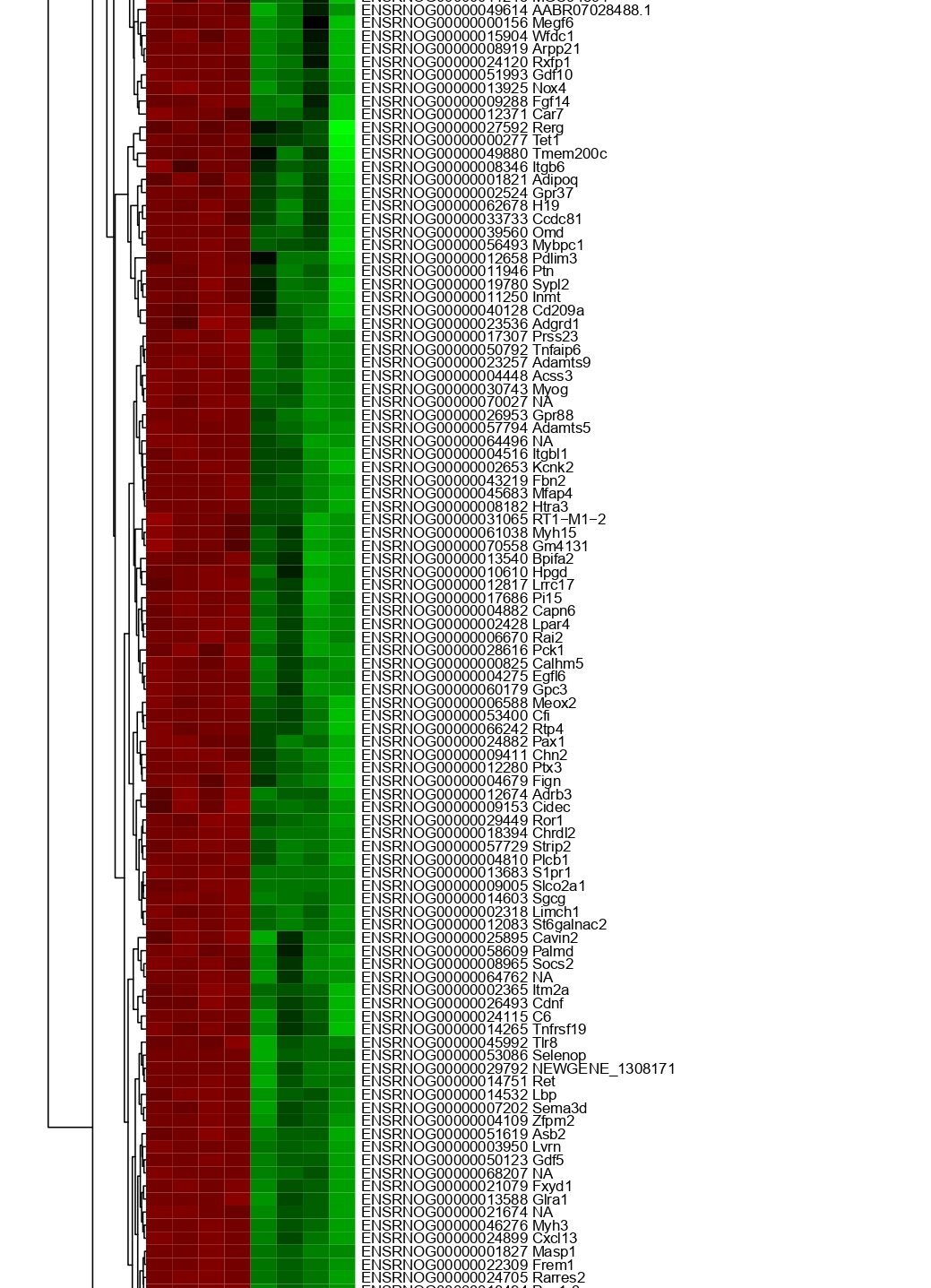

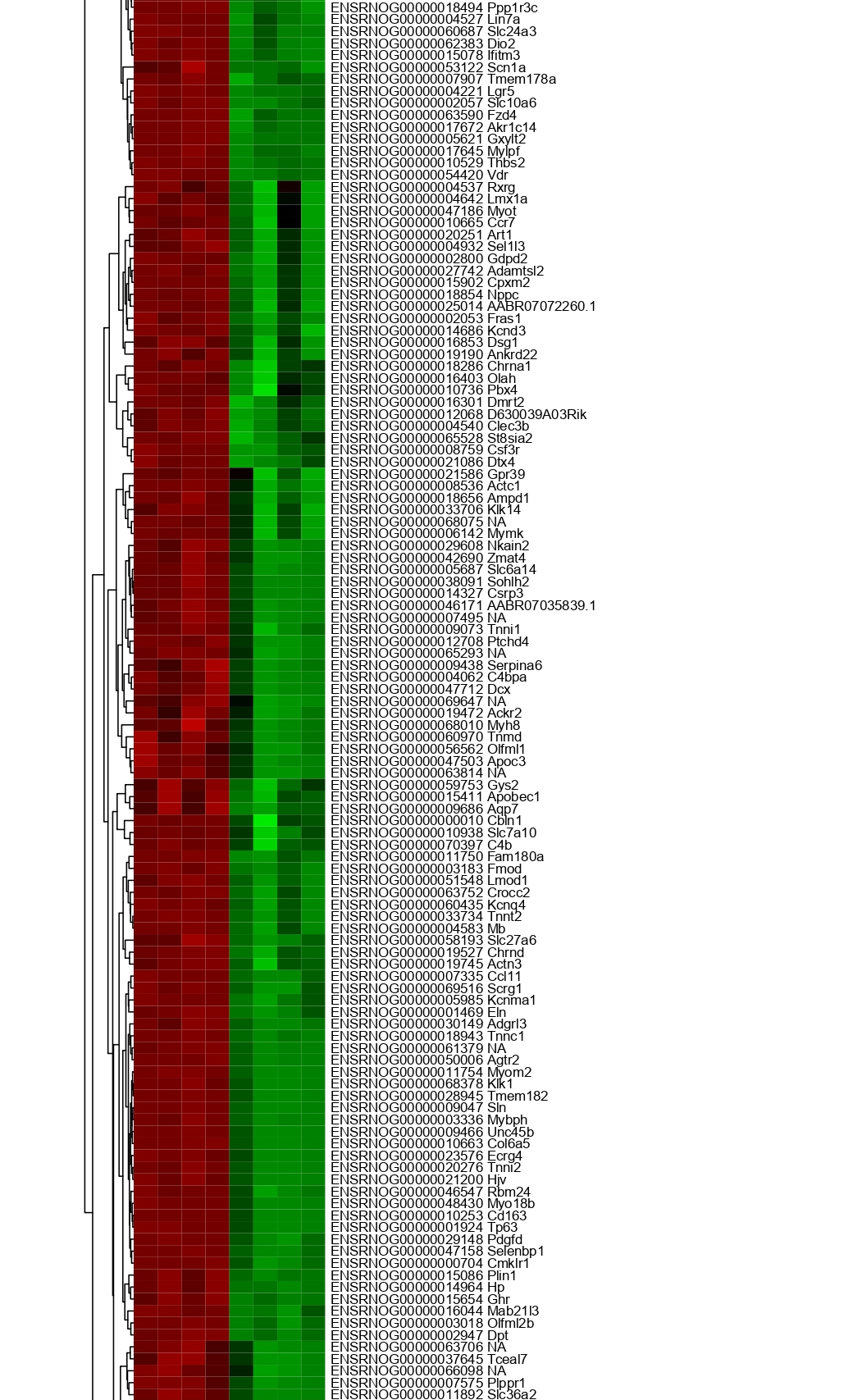

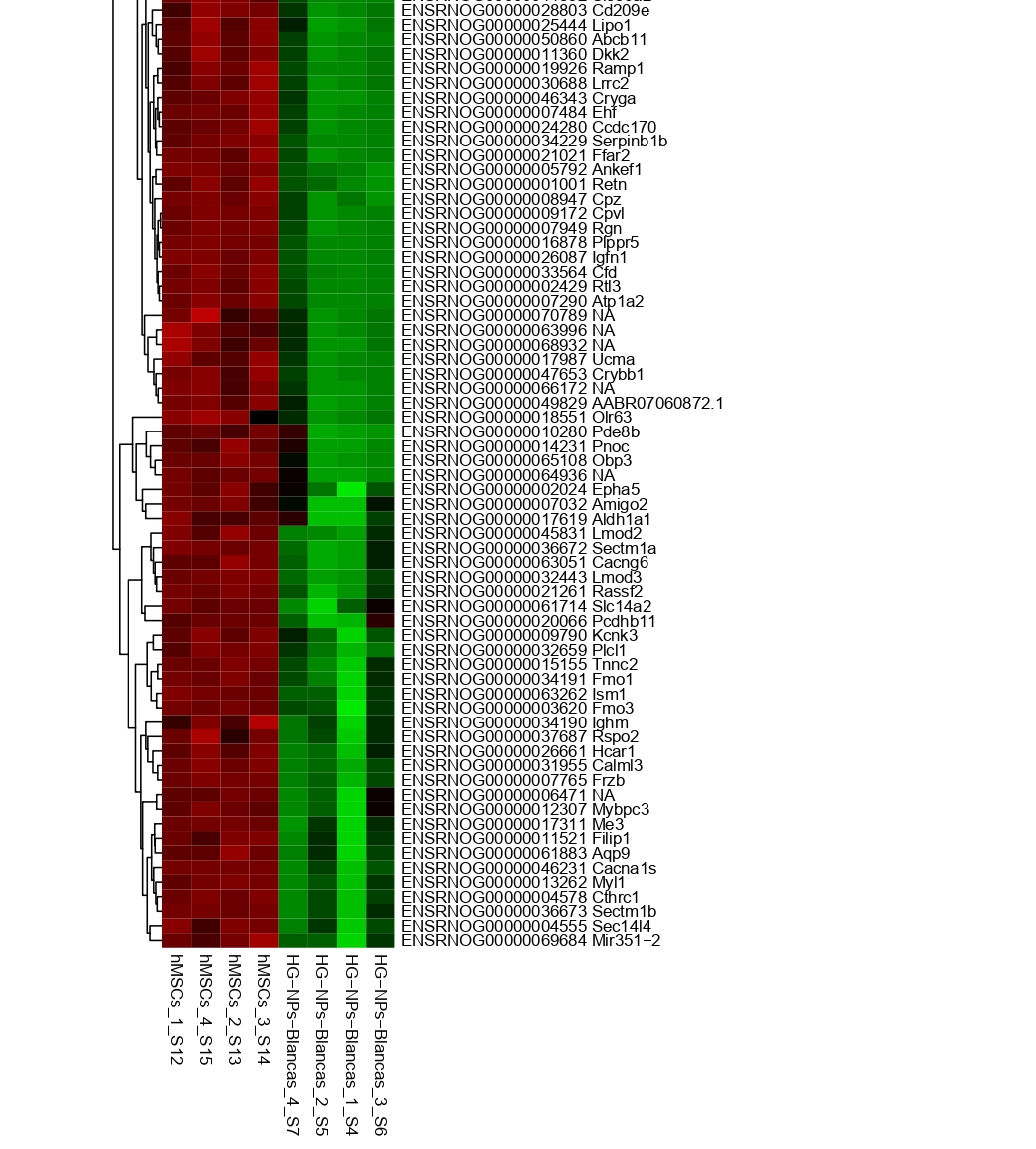


Figure S6. Heat map of differentially regulated genes due to the 3D environment provided by the nanoreinforced supramolecular HG. Comparison between 2D-cultured MSCs vs. MSCs cultured within the nanoreinforced HG.
